# Sodium butyrate inhibits high cholesterol-induced neuronal amyloidogenesis by modulating NRF2 stabilization-mediated ROS levels: involvement of NOX2 and SOD1

**DOI:** 10.1038/s41419-020-2663-1

**Published:** 2020-06-18

**Authors:** Seo Yihl Kim, Chang Woo Chae, Hyun Jik Lee, Young Hyun Jung, Gee Euhn Choi, Jun Sung Kim, Jae Ryong Lim, Joo Eun Lee, Ji Hyeon Cho, Hansoo Park, Changho Park, Ho Jae Han

**Affiliations:** 1 Department of Veterinary Physiology, College of Veterinary Medicine, Research Institute for Veterinary Science, and BK21 PLUS Program for Creative Veterinary Science Research, Seoul National University, Seoul, 08826 Republic of Korea; 2 Laboratory of Veterinary Physiology, College of Veterinary Medicine, Chungbuk National University, Cheongju, Chungbuk 28644 South Korea; 3 Institute for Stem Cell & Regenerative Medicine (ISCRM), Chungbuk National University, Cheongju, 28644 Chungbuk Korea; 4 Department of Biomedical Science and Engineering, Gwangju Institute of Science and Technology (GIST), Gwangju, Korea; 5 Genome and Company, Pangyo-ro 253, Bundang-gu. Seoungnam-si, Gyeonggi-do, 13486 Korea

**Keywords:** Cell signalling, Diseases of the nervous system

## Abstract

The gut–brain axis is currently being studied as a therapeutic strategy for neurological diseases, especially Alzheimer’s disease (AD). Obesity results in the gut microbiota dysbiosis, which includes butyrate-producing bacteria are reduced. Although sodium butyrate (NaB) has emerged as the potential therapeutic substance in AD, there is a lack of detailed results into what signaling pathways affect amyloidogenesis in AD induced by obesity. Thus, we investigated the regulatory role of NaB on amyloidogenesis in neuronal cells under high cholesterol. In our results, we verified that increased amyloid β peptide (Aβ) accumulation in the brain of obese mice and a reduction in butyrate-producing bacteria due to the gut microbiota dysbiosis induced by obesity. We showed that NaB decreased the expression levels of beta-site amyloid precursor protein cleaving enzyme 1 (BACE1) and Aβ accumulation induced by high cholesterol in SK-N-MC cells. We demonstrated that NaB was absorbed in cells through sodium-coupled monocarboxylate transporter 1 (SMCT1) and then inhibited high cholesterol-induced Aβ accumulation. Subsequently, we also observed that reactive oxygen species (ROS) were overproduced because of increased NADPH oxidase 2 (NOX2) expression under high cholesterol. Meanwhile, NaB decreased NOX2 levels through a reduction of NF-κB activity, which ultimately inhibited Aβ accumulation caused by high cholesterol. We demonstrated that NaB increased the expression levels of p21 under high cholesterol, contributing to p21/NRF2 (Nuclear factor erythroid 2-related factor 2) colocalization, which leads to NRF2 stabilization. NRF2 stabilization causes NF-κB inactivation, followed by NOX2 suppression and superoxide dismutase 1 (SOD1) upregulation. Thus, NaB with *SOD1* silencing under high cholesterol did not eliminate excessive ROS, and eventually resulted in Aβ accumulation. In conclusion, we demonstrated that NaB prevents excessive ROS through NOX2 suppression and SOD1 upregulation by p21/NRF2 pathway, which is critical for inhibiting BACE1-dependent amyloidogenesis in neuronal cells exposed to high cholesterol environment.

## Introduction

The influence of gut microbiota on central nervous system (CNS) function is called the “gut–brain axis”^1^. The gut–brain axis is emerging as a therapeutic strategy for neurological diseases, especially AD; thus, many studies are under way focusing on the alteration of gut microbiome and metabolites of gut microbiota to prevent AD^2–4^. Accumulating studies have shown that the gut microbiota composition in obese patients showed that butyrate-producing bacteria are reduced in the gut^5,6^. In previous studies, NaB was already proposed as a therapeutic substance for AD through antioxidant and anti-inflammatory functions^7,8^. However, there are few studies on how the mechanism of NaB regulates obesity-induced AD. Therefore, the study of how NaB affects amyloidogenesis caused by obesity is meaningful. NaB plays diverse biological roles, and it affects cell signaling pathways through mitochondrial activity regulation, histone deacetylase (HDAC) inhibitor, and G-protein coupled receptor (GPCR)^9–11^. However, the specific mechanisms by which NaB inhibits Aβ secretion in neurons remain still unclear. Therefore, it will be a key factor of the mechanism whether NaB is absorbed into neurons and acts as an HDAC inhibitor or affects cell signaling pathways as a GPCR ligand.

Many studies have shown various evidence that obesity induces AD, and obesity has been also reported as a risk factor that induces AD independently of diabetes mellitus^12–14^. One of typical characteristics found in obese patients is high-cholesterol levels, which is also a major cause of the pathogenesis of AD^12,15,16^. When cells are exposed to high-cholesterol environment, they produce excessive ROS^17^. Neurons under high cholesterol also produce excessive ROS, which is known as one of the major contributors to amyloidogenesis^18^. There are generally two types of ROS sources in the cells; one is NOX and the other is mitochondria^19^. Previous studies reported that the critical factor of ROS in the CNS is NOX^20^, but further studies are needed to determine the specific source of excessive ROS produced in neurons under high cholesterol. Thus, it is essential to investigate the major source of excessive ROS caused by high cholesterol and how NaB affects cell signaling pathways associated with NOX or mitochondrial ROS (mtROS).

The most prominent transcription factor that regulates expression of antioxidant enzymes is an NRF2^21^. In response to oxidative damages, cells are protected by NRF2/Keap1/ARE antioxidant pathway^21,22^. However, a study reported that overproduced ROS in response to oxidative damages make antioxidant defense vulnerable^23^. Therefore, since NaB has been shown to act as an activator of NRF2^24^, the mechanism by which NaB activates NRF2 under high cholesterol will be meaningful. Previous studies reported that NaB increases p21 levels, which contributes to NRF2 stabilization by binding to NRF2^25,26^. Therefore, it seems necessary to investigate the specific mechanism by which NaB stabilizes NRF2 through p21/NRF2 pathway.

In the present study, we prepared a high fat diet (HFD)-induced mice models of obesity for AD because HFD feeding has been reported to cause cognitive impairment^27–29^. To investigate the exact mechanisms of the inhibitory effect of NaB on amyloidogenesis, we used SK-N-MC cells, which are a human neuronal epithelioma cell line^30^. SK-N-MC cells are well-stabilized cell line and widely used as a model for studying cellular signal transduction in many AD studies^31,32^. To address this issue, we identified the mechanisms of how NaB reduces high cholesterol-induced ROS and investigated the protective role of NaB against amyloidogenesis caused by high cholesterol.

## Result

### BACE1 expression and Aβ accumulation in the brain of the obesity model and the alteration of the gut microbiota composition in the obesity model

**Fig. 1:**
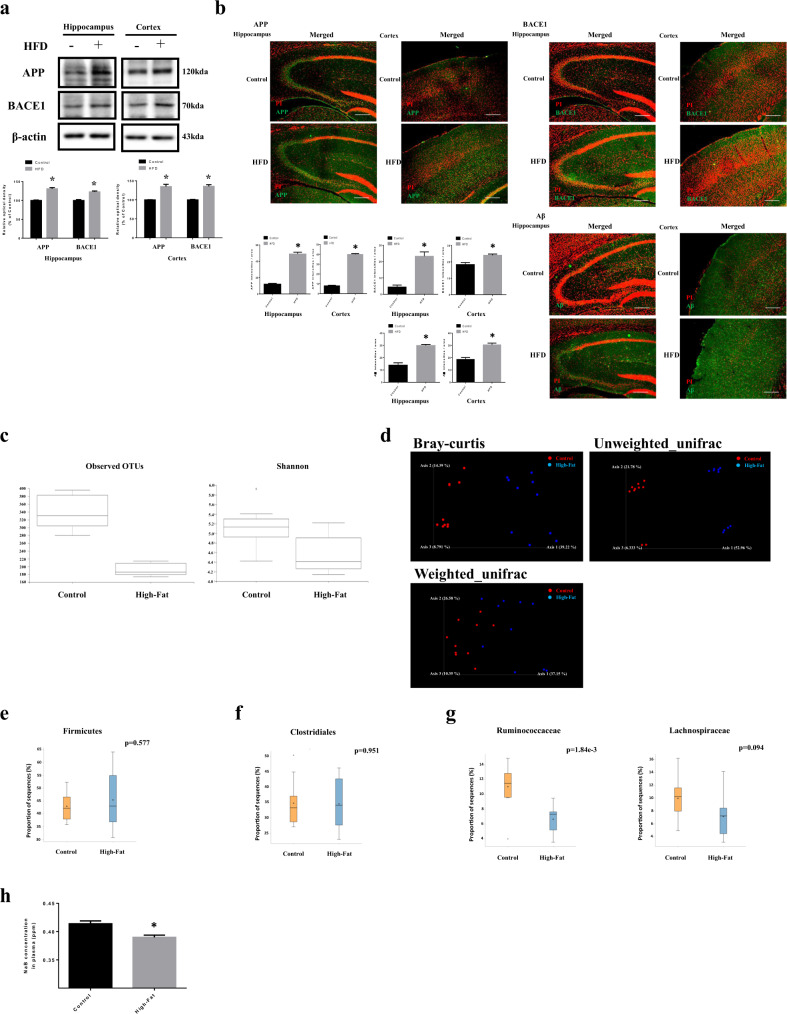
BACE1 expression and Aβ accumulation in the brain of the obesity model and the alteration of the gut microbiota composition in the obesity model. **a** Brains of 20 weeks old ICR mice, which were fed with HFD for 14 weeks were extracted. The extracted brains were cryo-sectioned coronally, and separated into hippocampus and cortex. The expression levels of APP, BACE1, and β-actin were analyzed by western blot. β-actin was used as a loading control. Data are presented as a mean ± S.E.M. *n* = 4. **b** Brain samples for immunohistochemistry were immunostained with APP, BACE1, and Aβ and PI. Images shown in result are representative. All scale bars, 25 µm (magnification, ×100) **c** Alpha diversity between control and high-fat; left panel: Observed OTUs, right panel: Shannon’s diversity index. **d** Beta diversity between control (•) and high-fat (•); Bray–Curtis, unweighted UniFrac distance, weighted UniFrac distance. **e**–**g** STAMP analysis; **e** Differences in relative abundance of firmicutes between groups at the phylum level, **f** Differences in relative abundance of clostridiales between groups at the order level, **g** Differences in relative abundance of ruminococcaceae and lachnospiraceae between groups at the family level. **h** The concentration of NaB in plasma of mice was measured by GC–MS. NaB concentration data are presented as a mean ± S.E.M. *n* = 4. *p < 0.05 versus control. All blot and immunofluorescence images shown are representative.

We verified BACE1 expression and Aβ accumulation in the hippocampus and cortex to confirm the effect of obesity. We found that amyloid precursor protein (APP) and BACE1 levels were higher in HFD-fed mice than in normal diet (ND)-fed mice (Fig. 1a). In immunohistochemistry results, APP, BACE1, and Aβ were also increased in the hippocampus and cortex in brains of HFD-fed mice (Fig. 1b). The analysis of gut microbiota diversity mainly includes alpha diversity and beta diversity. In brief, alpha diversity identifies the distribution of various microorganisms in a sample and beta diversity is a method of checking whether the sample diversity is similar^33,34^. The analyses showed that the gut microbiota diversity was lower in HFD-fed mice than in ND-fed mice (Fig. 1c, d). Firmicutes to which most butyrate-producing bacteria belong in phylum level were not altered in the HFD group compared to the ND group (Fig. 1e). Clostridiales to which most butyrate-producing bacteria belong in order level were also not changed in the HFD group (Fig. 1f). In the case of Ruminococcaceae and Lachnospiraceae to which butyrate-producing bacteria belong in family level^35^, there was a significant reduction in HFD-fed mice (Fig. 1g). We presented the list of *p*-value in phylum, order, and family level of gut microbiota in the obesity model in Supplementary Table S1. Furthermore, we measured concentration of NaB in plasma from mice by using gas chromatography–mass spectrometry (GC–MS). The concentration of NaB in plasma from HFD-fed mice was lower than ND-fed mice (Fig. 1h). Subsequently, we also measured high cholesterol levels in plasma from mice. Our data showed that total cholesterol levels were significantly higher in HFD-fed mice [Supplementary Fig. S1].

### Effect of NaB on high cholesterol-induced BACE1 expression and Aβ accumulation

**Fig. 2:**
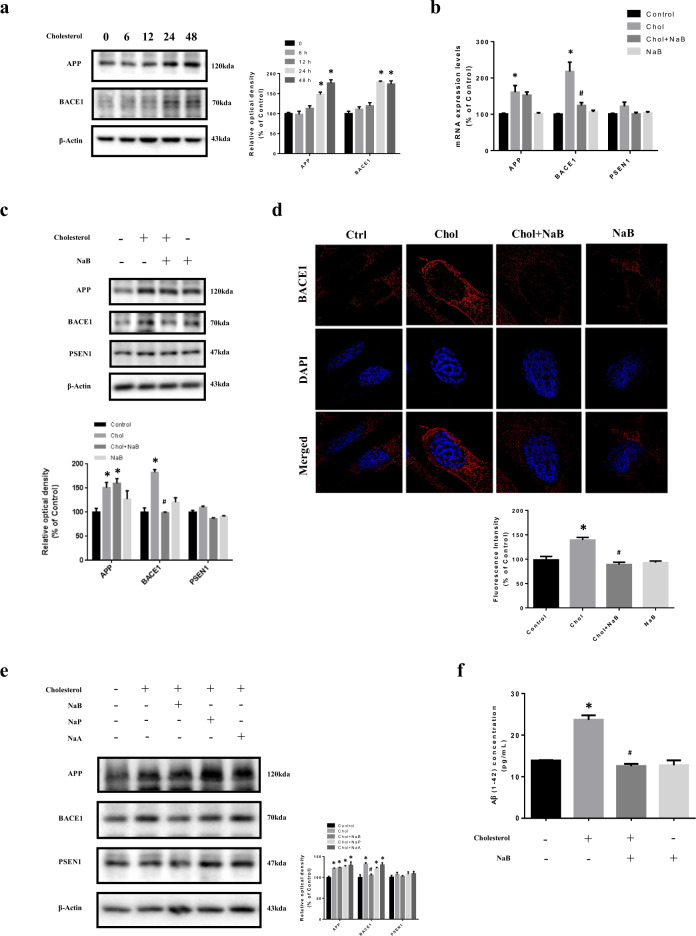
Effect of NaB on high-cholesterol-induced BACE1 expression and Aβ accumulation. **a** SK-N-MC cells were treated with high cholesterol (25 µM) for various time (0–48 h). APP and BACE1 were analyzed by western blot. β-actin was used as a loading control. *n* = 4. **b** Cells were pretreated with NaB (500 µM) for 30 min prior to treatment of high cholesterol for 24 h. The mRNA expression levels of *APP, BACE1, and PSEN1* were analyzed by quantitative real-time PCR. Data were normalized by the *ACTB* mRNA expression levels. *n* = 4. **c** Cells were pretreated with NaB for 30 min prior to treatment of high cholesterol for 24 h. The expression levels of APP, BACE1, and PSEN1 were analyzed by western blot. β-actin was used as a loading control. *n* = 4. **d** Cells were pretreated with NaB for 30 min prior to treatment of high cholesterol for 24 h and immunostained with BACE1 antibody. Scale bars are 8 µm (magnification, ×1,000). *n* = 3. **e** Cells were pretreated with NaB, NaP (500 µM), and NaA (500 µM) for 30 min prior to treatment of high cholesterol for 24 h. The expression levels of APP, BACE1, and PSEN1 were analyzed by western blot. β-actin was used as a loading control. *n* = 3. **f** Cells were pretreated with NaB for 30 min prior to treatment for high cholesterol for 72 h. Aβ concentration of medium samples was detected by using ELISA kit. Data are presented as a mean ± S.E.M. *n* = 4. *p < 0.05 versus control, ^#^*p* < 0.05 versus high-cholesterol treatment. All blot and immunofluorescence images shown are representative.

To confirm effect of high cholesterol on Aβ, we analyzed APP and BACE1 levels in SK-N-MC cells treated with high cholesterol for 0 to 48 h. High cholesterol increased APP and BACE1 levels at 24–48 h (Fig. 2a). We investigated whether NaB affects APP, BACE1, and presenilin-1 (PSEN1) levels. Our data showed that NaB decreased only high cholesterol-induced BACE1 (Fig. 2b, c). In immunofluorescence staining results, the fluorescence intensities of BACE1 under high cholesterol were increased, but recovered by NaB (Fig. 2d). These results indicated that Aβ secretion induced by high cholesterol was dependent on BACE1, and NaB decreased BACE1 levels, which eventually blocked Aβ accumulation. We also performed *BACE1* siRNA transfection to verify that Aβ secretion caused by high cholesterol depends on BACE1. Our data showed that Aβ levels were decreased by *BACE1* siRNA transfection under high cholesterol [Supplementary Fig. S2]. Next, we compared effect of short chain fatty acids (SCFAs) on APP, BACE1, and PSEN1 levels. Sodium propionate (NaP) and sodium acetate (NaA) did not significantly affect anything, but NaB influenced only BACE1 levels (Fig. 2e). In addition, when Aβ levels were measured by enzyme-linked immunosorbent assay (ELISA), the levels treated with NaB under high cholesterol were decreased (Fig. 2f).

### Involvement of SMCT1 in inhibitory effect of NaB on high cholesterol-induced ROS generation, BACE1 expression, and Aβ accumulation

**Fig. 3:**
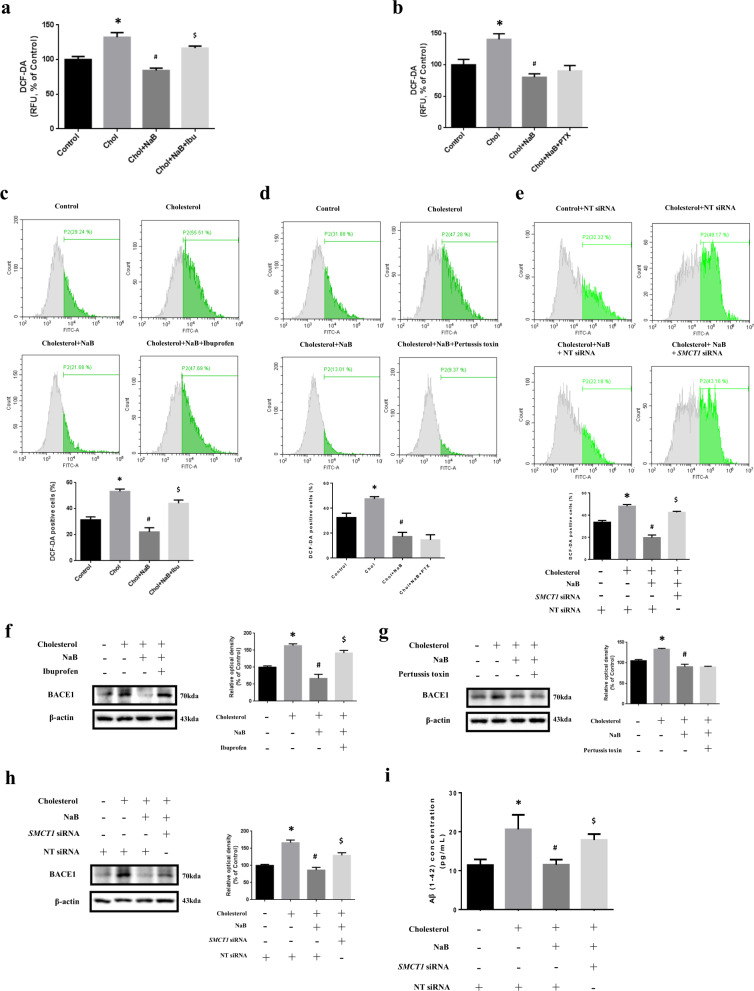
Involvement of SMCT1 in inhibitory effect of NaB on high cholesterol-induced ROS generation, BACE1 expression, and Aβ accumulation. **a** SK-N-MC cells were pretreated with NaB and ibuprofen (500 µM) for 30 min prior to treatment of high cholesterol for 48 h where DCF-DA was detected by luminometer. *n* = 4. *p < 0.05 versus control, ^#^*p* < 0.05 versus high cholesterol treatment, ^$^*p* < 0.05 versus treatment of high cholesterol and NaB. **b** Cells were pretreated with NaB and PTX (200 nM) for 30 min prior to treatment of high cholesterol for 48 h where DCF-DA was detected by luminometer. *p < 0.05 versus control, ^#^*p* < 0^.^05 versus high cholesterol treatment. **c** Cells were pretreated with NaB and ibuprofen for 30 min prior to treatment of high cholesterol for 72 h where ROS with DCF-DA were measured by flowcytometer. Total cell counts = 1.0 × 10^4^ cells. *n* = 4. *p < 0.05 versus control, ^#^*p* < 0.05 versus high cholesterol treatment, ^$^*p* < 0.05 versus treatment of high cholesterol and NaB. **d** Cells were pretreated with NaB and PTX for 30 min prior to treatment of high cholesterol for 72 h where ROS with DCF-DA were measured by flowcytometer. Total cell counts = 1.0 × 10^4^ cells. *n* = 4. *p < 0.05 versus control, ^#^*p* < 0.05 versus high cholesterol treatment. **e** Cells were transfected with *SMCT1* siRNA or NT siRNA for 12 h, and pretreated with NaB for 30 min prior to treatment of high cholesterol for 72 h where ROS with DCF-DA were measured by flowcytometer. Total cell counts = 1.0 × 10^4^ cells. Data are presented as a mean ± S.E.M. *n* = 3. *p < 0.05 versus control with NT siRNA transfection, ^#^*p* < 0.05 versus high cholesterol treatment with NT siRNA transfection, ^$^*p* < 0.05 versus treatment of high cholesterol and NaB with NT siRNA transfection. **f** Cells were pretreated with NaB and ibuprofen for 30 min prior to treatment of high cholesterol for 24 h. The expression levels of BACE1 were analyzed by western blot. β-actin was used as a loading control. *n* = 3. *p < 0.05 versus control, ^#^*p* < 0.05 versus high cholesterol treatment, ^$^*p* < 0.05 versus treatment of high cholesterol and NaB. **g** Cells were pretreated with NaB and PTX for 30 min prior to treatment of high cholesterol for 24 h. The expression levels of BACE1 were analyzed by western blot. β-actin was used as a loading control. *n* = 3. *p < 0.05 versus control, ^#^*p* < 0.05 versus high-cholesterol treatment. **h** Cells were transfected with *SMCT1* siRNA or NT siRNA for 12 h, and pretreated with NaB for 30 min prior to treatment of high cholesterol for 24 h. The expression levels of BACE1 were analyzed by western blot. β-actin was used as a loading control. *n* = 3. *p < 0.05 versus control with NT siRNA transfection, ^#^*p* < 0.05 versus high cholesterol treatment with NT siRNA transfection, ^$^*p* < 0.05 versus treatment of high cholesterol and NaB with NT siRNA transfection. **i** Cells were transfected with *SMCT1* siRNA or NT siRNA for 12 h, and pretreated with NaB for 30 min prior to treatment of high cholesterol for 72 h. Aβ concentration of medium samples was detected by using ELISA kit. Data are presented as a mean ± S.E.M. *n* = 4. *p < 0.05 versus control with NT siRNA transfection, ^#^*p* < 0.05 versus high cholesterol treatment with NT siRNA transfection, ^$^*p* < 0.05 versus treatment of high cholesterol and NaB with NT siRNA transfection. All blot images shown are representative.

We investigated whether the reason for a reduction of high cholesterol-induced ROS by NaB is through acting as an HDAC inhibitor or as a GPCR ligand. In our data, high cholesterol-induced ROS were reduced by NaB, but when both NaB and ibuprofen (SMCT inhibitor) were pretreated under high cholesterol, ROS were not decreased (Fig. 3a). However, when pertussis toxin (PTX, Gαi inhibitor) was pretreated instead of ibuprofen, ROS caused by high cholesterol were decreased (Fig. 3b). Consistent with Fig. 3a, b, Our data showed that excessive ROS were not reduced by NaB and ibuprofen, but by NaB and PTX by using flow cytometry (Fig. 3c, d). However, there is a study that ibuprofen has an antioxidant effect^36^. Thus, we performed *SMCT1* siRNA transfection: the ratio of SMCT1 is high in neurons^37^. In our results, high-cholesterol-induced ROS were reduced by NT siRNA transfection and NaB, but *SMCT1* siRNA transfection and NaB led to ROS accumulation (Fig. 3e). Furthermore, BACE1 levels were decreased by NaB, and increased when both NaB and ibuprofen were pretreated under high cholesterol (Fig. 3f). In contrast, when PTX was pretreated with NaB, BACE1 levels were decreased under high cholesterol (Fig. 3g). In addition, BACE1 and Aβ levels were not decreased by *SMCT1* siRNA transfection and NaB under high cholesterol (Fig. 3h, i).

### Effect of NaB on high cholesterol-induced NOX2 expression

**Fig. 4:**
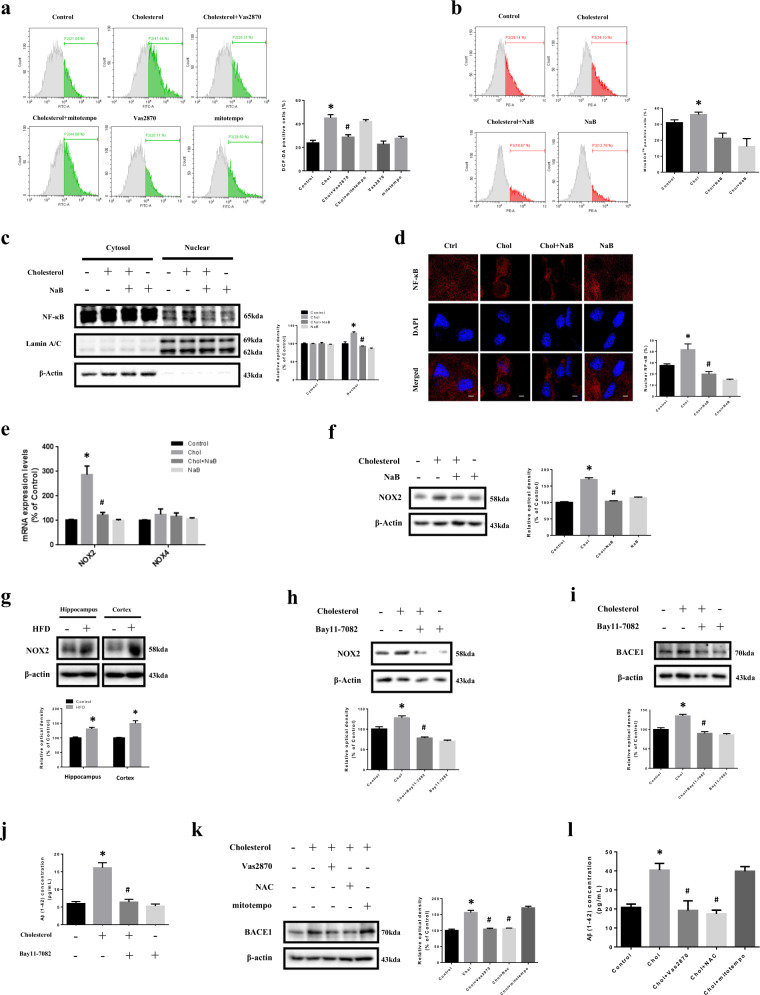
Effect of NaB on high cholesterol-induced NOX2 expression. **a** SK-N-MC cells were pretreated with Vas2870 (5 µM) or mitotempo (2 µM) for 30 min prior to high cholesterol treatment for 72 h where ROS with DCF-DA were measured by flowcytometer. Total cell counts = 1.0 × 10^4^ cells. *n* = 4. *p < 0.05 versus control, ^#^*p* < 0.05 versus high cholesterol treatment. **b** Cells were pretreated with NaB for 30 min prior to high cholesterol treatment for 72 h where mtROS with mitoSox^TM^ red were measured by flowcytometer. Total cell counts = 1.0 × 10^4^ cells. *n* = 4. ^#^*p* < 0.05 versus high cholesterol treatment. **c** Cells were pretreated with NaB for 30 min prior to high cholesterol treatment for 24 h. NF-κB, Lamin A/C, and β-actin protein levels in cytosolic and nuclear-fractionized samples were analyzed by western blot. *n* = 3. *p < 0.05 versus control, ^#^*p* < 0^.^05 versus high cholesterol treatment. **d** Cells were pretreated with NaB for 30 min prior to high cholesterol treatment for 24 h and immunostained with NF-κB antibody. Scale bars are 8 µm (magnification, ×1,000). *n* = 3. *p < 0.05 versus control, ^#^*p* < 0.05 versus high cholesterol treatment. **e** Cells were pretreated with NaB for 30 min prior to high cholesterol treatment for 24 h. Data were normalized by the *ACTB* mRNA expression levels. *n* = 4. **f** Cells were pretreated with NaB for 30 min prior to high cholesterol treatment for 24 h. The expression levels of NOX2 were analyzed by western blot. β-actin was used as a loading control. *n* = 4. *p < 0.05 versus control, ^#^*p* < 0.05 versus high cholesterol treatment. **g** Brains of 20 weeks mice fed with HFD for 14 weeks were extracted. The expression levels of NOX2 were analyzed by western blot. β-actin was used as a loading control. *n* = 3. *p < 0.05 versus control. **h**, **i** Cells were pretreated with Bay11-7082 (5 µM) for 30 min prior to high-cholesterol treatment for 24 h. The expression levels of NOX2 and BACE1 were analyzed by western blot. β-actin was used as a loading control. *n* = 4. **j** Cells were pretreated with Bay11-7082 for 30 min prior to high cholesterol treatment for 72 h. Aβ concentration of medium samples was detected by using ELISA kit. *n* = 4. **k** Cells were pretreated with Vas2870 or NAC (5 mM) or mitotempo for 30 min prior to high-cholesterol treatment for 24 h. The expression levels of BACE1 were analyzed by western blot. β-actin was used as a loading control. *n* = 4. **l** Cells were pretreated with Vas2870 or NAC or mitotempo for 30 min prior to high-cholesterol treatment for 72 h. Aβ concentration of medium samples was detected by using ELISA kit. Data are presented as a mean ± S.E.M. *n* = 4. *p < 0.05 versus control, ^#^*p* < 0.05 versus high cholesterol treatment. All blot and immunofluorescence images shown are representative.

To determine main source of ROS produced by high cholesterol, we pretreated Vas2870 (NOX inhibitor) and mitotempo (mtROS inhibitor). High cholesterol-induced ROS were blocked by Vas2870 but not by mitotempo (Fig. 4a). We also checked mtROS by staining with MitoSOX^TM^ Red. In our results, mtROS did not show a significant increase (Fig. 4b). Previous studies reported that the most prominent transcription factor of NOX2 is nuclear factor-kappa B (NF-κB)^38^. Our results showed that NF-κB levels in the nucleus were increased by high cholesterol, but decreased by NaB (Fig. 4c, d). Previous studies reported that NOX1-4 are major isotypes of NOX expressed in the brain, and there seems to be difference in expression depending on cell types^39,40^. Therefore, we made primers referring to previous studies and gradient PCR was performed with complementary DNA of SK-N-MC cells^41,42^. Our results showed that *NOX1* and *NOX3* were hardly expressed in the cells [Supplementary Fig. S3], so real-time PCR was performed with *NOX2* and *NOX4*. In our results, mRNA expression levels of *NOX2* showed greater increase by high cholesterol but decreased by NaB (Fig. 4e). Consistently, protein levels of NOX2 were increased by high cholesterol but decreased by NaB (Fig. 4f). We also showed NOX2 levels in the hippocampus and cortex and found that NOX2 levels were higher in HFD-fed mice (Fig. 4g). We further investigated whether NF-κB is a critical transcription factor for NOX2. The increased NOX2 levels under high cholesterol were decreased by Bay11-7082 (NF-κB inhibitor) (Fig. 4h). Subsequently, increased BACE1 levels and Aβ secretion induced by high cholesterol were decreased by Bay11-7082 (Fig. 4i, j). We further confirmed the involvement of NF-κB in high cholesterol-induced ROS, NOX2, BACE1, and Aβ through Bay11-7082 and *p65* siRNA transfection. Our data showed that both Bay11-7082 and *p65* knockdown decreased ROS, NOX2 and BACE1 levels, and Aβ accumulation caused by high cholesterol [Supplementary Fig. S4]. In our results, increased BACE1 and Aβ levels by high cholesterol were decreased by Vas2870 and N-acetylcysteine (NAC; ROS scavenger), but not mitotempo (Fig. 4k, l). Finally, to further confirm involvement of NOX2 in high cholesterol-induced BACE1 and Aβ, we performed *NOX2* siRNA transfection. Our results showed that BACE1 and Aβ levels were decreased by *NOX2* siRNA transfection under high cholesterol [Supplementary Fig. S5].

### Regulatory role of NaB in high cholesterol-induced downregulation of p21 and p21/NRF2 colocalization

**Fig. 5:**
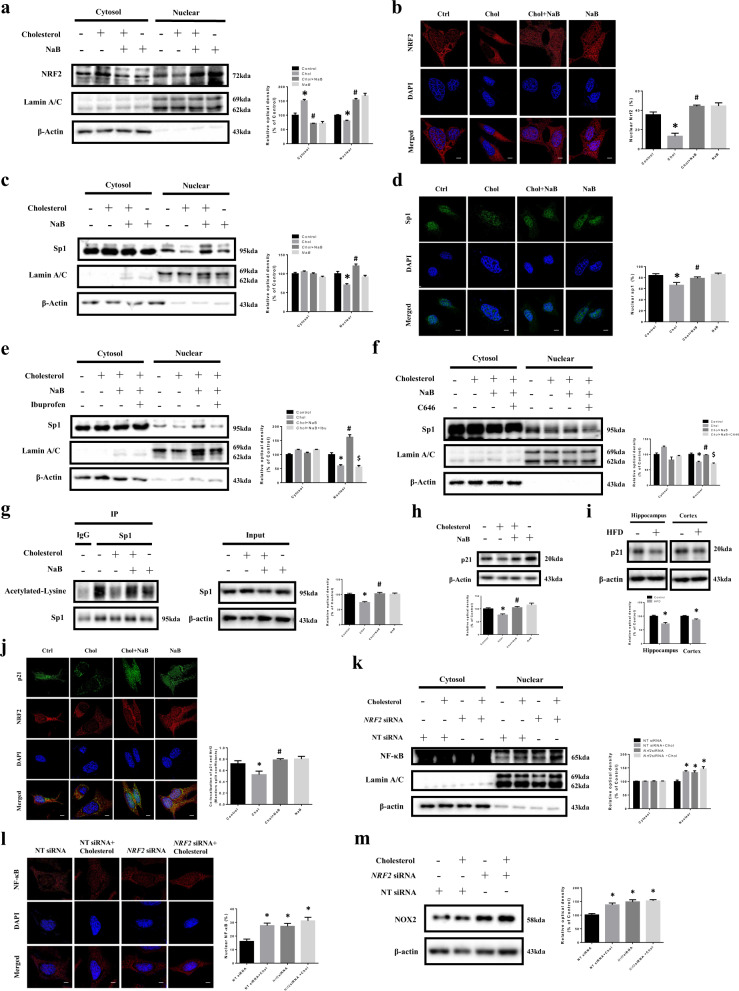
Regulatory role of NaB in high cholesterol-induced downregulation of p21 and p21/NRF2 colocalization. **a** SK-N-MC cells were pretreated with NaB for 30 min prior to high cholesterol treatment for 24 h. NRF2, Lamin A/C, and β-actin protein levels in cytosolic and nuclear-fractionized samples were analyzed by western blot. *n* = 3. **b** Cells were immunostained with NRF2 antibody. Scale bars are 8 µm (magnification, ×1,000). *n* = 3. **c** SK-N-MC cells were pretreated with NaB for 30 min prior to high cholesterol treatment for 24 h. Sp1, Lamin A/C, and β-actin protein levels in cytosolic and nuclear-fractionized samples were analyzed by western blot. *n* = 3. **d** Cells were immunostained with sp1 antibody. Scale bars are 8 µm (magnification, ×1,000). *n* = 4. *p < 0.05 versus control, ^#^*p* < 0.05 versus high cholesterol treatment. **e** Cells were pretreated with NaB and ibuprofen for 30 min prior to treatment of high cholesterol for 24 h. Sp1, Lamin A/C, and β-actin protein levels in cytosolic and nuclear-fractionized samples were analyzed by western blot. *n* = 3. **f** Cells were pretreated with NaB and C646 for 30 min prior to treatment of high cholesterol for 24 h. Sp1, Lamin A/C, and β-actin protein levels in cytosolic and nuclear-fractionized samples were analyzed by western blot. *n* = 3. *p < 0.05 versus control, ^#^*p* < 0.05 versus high cholesterol treatment, ^$^*p* < 0.05 versus treatment of high cholesterol and NaB. **g** Cells were pretreated with NaB for 30 min prior to high cholesterol treatment for 24 h. Co-immunoprecipitation of acetylated-lysine with IgG and Sp1 was shown in left panel. Total protein expressions in lysate were shown in right panel. *n* = 3. **h** Cells were pretreated with NaB for 30 min prior to high cholesterol treatment for 24 h. The expression levels of p21 were analyzed by western blot. β-actin was used as a loading control. *p < 0.05 versus control, ^#^*p* < 0.05 versus high-cholesterol treatment. **i** Brains of 20 weeks mice fed with HFD for 14 weeks were extracted. The expression levels of p21 were analyzed by western blot. β-actin was used as a loading control. *n* = 3. *p < 0.05 versus control. **j** Cells were pretreated with NaB for 30 mi*n* prior to high cholesterol treatment for 24 h and immunostained with p21 and NRF2 antibodies. Colocalization of p21 (green) and NRF2 (red) was visualized with SRRF imaging system. Scale bars are 8 µm (magnification, ×1,000). *n* = 4. *p < 0.05 versus control, ^#^*p* < 0.05 versus high cholesterol treatment. **k** Cells were transfected with *NRF2* siRNA or NT siRNA for 12 h, and treated with high cholesterol for 24 h. NF-κB, Lamin A/C, and β-actin protein levels in cytosolic and nuclear-fractionized samples were analyzed by western blot. *n* = 3. **l** Cells were immunostained with NF-κB antibody. Scale bars are 8 µm (magnification, ×1,000). *n* = 4. **m** Cells were transfected with *NRF2* siRNA or NT siRNA for 12 h, and treated with high cholesterol for 24 h. The expression levels of NOX2 were analyzed by western blot. β-actin was used as a loading control. Data are presented as a mean ± S.E.M. *n* = 4. *p < 0.05 versus NT siRNA transfection. All blot and immunofluorescence images shown are representative.

We investigated the effect of NaB on the nuclear translocation of NRF2 under high cholesterol. NRF2 levels in the nucleus were decreased by high cholesterol, but were increased by NaB (Fig. 5a, b). In our results, activity of specificity protein 1 (Sp1), which is one of the most prominent transcription factors of p21^43^, was decreased under high cholesterol, but recovered by NaB (Fig. 5c, d). Previous study has shown that acetylation of transcription factors affects the nuclear translocation^44^. Therefore, we verified whether the acetylation of Sp1 facilitates the nuclear translocation of sp1. Pretreatment of ibuprofen and NaB under high cholesterol resulted in reduction of sp1 nuclear translocation (Fig. 5e). We also confirmed the sp1 activation through acetylation of Sp1 by pretreating C646 (p300/CBP inhibitor) which inhibits Sp1 acetylation. In our results, nuclear translocation of Sp1 was decreased after pretreating with C646 and NaB under high cholesterol (Fig. 5f), and we finally verified the regulatory role of NaB on Sp1 acetylation under high cholesterol. Our immunoprecipitation data showed that the acetylation of sp1 reduced by high cholesterol was recovered by NaB (Fig. 5g). The data showed that p21 levels were decreased by high cholesterol, but were recovered by NaB (Fig. 5h). We further showed p21 levels in the hippocampus and cortex and found that p21 levels were lower in HFD-fed mice (Fig. 5i). Subsequently, we presented that colocalization of p21 and NRF2 was decreased by high cholesterol, but increased by NaB (Fig. 5j). We further presented results which showed the effect of NaB via NRF2, Sp1, and p21 on increased ROS, BACE1, and Aβ caused by high cholesterol [Supplementary Fig. S6–8]. There are studies demonstrating the crosstalk between NRF2 and NF-κB^45,46^. Therefore, we confirmed whether the nuclear translocation of NF-κB was increased by *NRF2* siRNA transfection. Our results showed that NF-κB levels in the nucleus were increased by *NRF2* siRNA transfection (Fig. 5k, l) and NOX2 levels were also increased by *NRF2* siRNA transfection under high cholesterol (Fig. 5m).

### Effect of NaB on high cholesterol-induced ROS generation, BACE1 expression, and Aβ accumulation through SOD1 expression

**Fig. 6:**
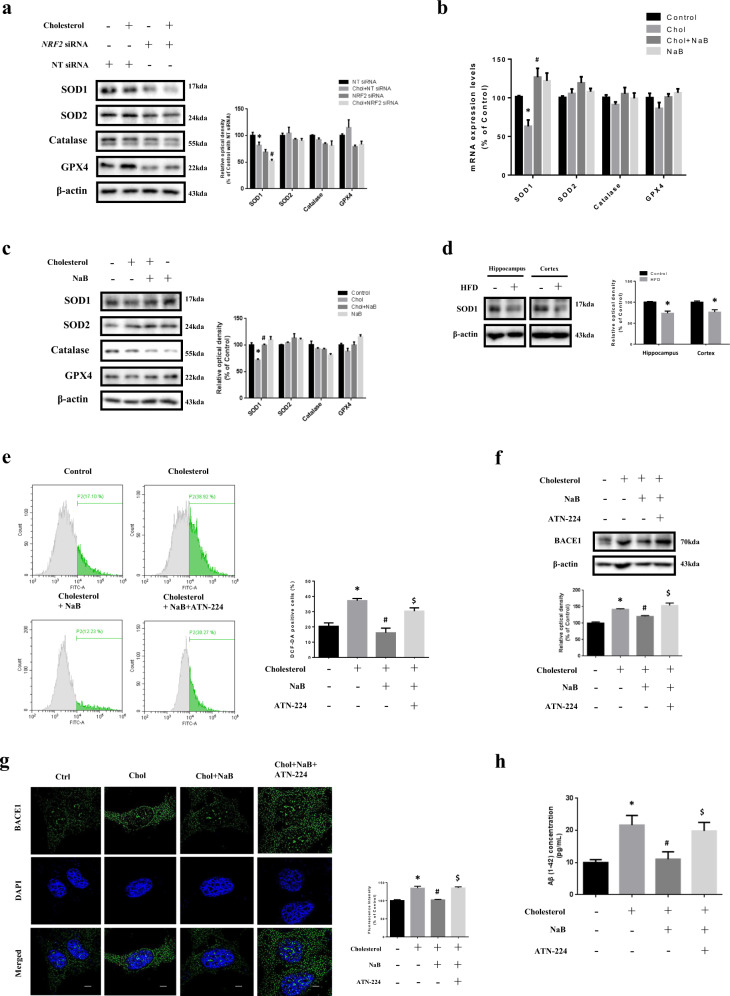
Effect of NaB on high-cholesterol-induced ROS generation, BACE1 expression, and Aβ accumulation through SOD1 expression. **a** SK-N-MC cells were transfected with *NRF2* siRNA or NT siRNA for 24 h, and pretreated with NaB for 30 min prior to treatment of high cholesterol for 24 h. The expression levels of SOD1, SOD2, catalase, and GPX4 were analyzed by western blot. β-actin was used as a loading control. *n* = 3. *p < 0.05 versus control with NT siRNA transfection, ^#^*p* < 0.05 versus high cholesterol treatment with NT siRNA transfection. **b** Cells were pretreated with NaB for 30 min prior to treatment of high cholesterol for 24 h. mRNA expression levels of *SOD1, SOD2, catalase*, and *GPX4* were analyzed by quantitative real-time PCR. Data were normalized by the *ACTB* mRNA expression levels. *n* = 4. *p < 0.05 versus control, ^#^*p* < 0.05 versus high cholesterol treatment. **c** Cells were pretreated with NaB for 30 min prior to treatment of high cholesterol for 24 h. The expression levels of SOD1, SOD2, catalase, and GPX4 were analyzed by western blot. β-actin was used as a loading control. *n* = 4. *p < 0.05 versus control, ^#^*p* < 0.05 versus high cholesterol treatment. **d** Brains of 20 weeks mice fed with HFD for 14 weeks were extracted. The expression levels of SOD1 were analyzed by western blot. β-actin was used as a loading control. *n* = 3. *p < 0.05 versus control. **e** Cells were pretreated with NaB and ATN^-^224 (4 µM) for 30 min prior to treatment of high cholesterol for 72 h where ROS with DCF-DA was measured by flowcytometer. Total cell counts = 1.0 × 10^4^ cells. *n* = 4. **f** Cells were pretreated with NaB and ATN-224 for 30 min prior to treatment of high cholesterol for 24 h. The expression levels of BACE1 were analyzed by western blot. β-actin was used as a loading control. *n* = 4. **g** Cells were pretreated with NaB and ATN-224 for 30 min prior to treatment of high cholesterol for 24 h and immunostained with BACE1 antibody. Scale bars are 8 µm (magnification, × 1,000). *n* = 3. H Cells were pretreated with NaB and ATN-224 for 30 min prior to treatment of high cholesterol for 72 h. Aβ concentration of medium samples was detected by using ELISA kit. Data are presented as a mean ± S.E.M. *n* = 4. *p < 0.05 versus control, ^#^*p* < 0.05 versus high cholesterol treatment, ^$^*p* < 0^.^05 versus treatment of high cholesterol and NaB. All blot and immunofluorescence images shown are representative.

We investigated that certain antioxidant enzymes which are products of NRF2 were significantly changed when treated with high cholesterol. Our results showed that only SOD1 levels were decreased by high cholesterol and *NRF2* silencing further decreased SOD1 levels under high cholesterol, but the others were not significantly altered (Fig. 6a). We also investigated antioxidant enzymes whose expression is suppressed under high cholesterol and recovered by NaB. Consistent with Fig. 6a, SOD1 levels were decreased by high cholesterol, but recovered by NaB while the others were not significantly changed (Fig. 6b, c). We also showed SOD1 levels in the hippocampus and cortex and found that SOD1 levels were lower in HFD-fed mice (Fig. 6d). To determine whether NaB removes excessive ROS through SOD1, ATN-224 (SOD1 inhibitor) was pretreated with NaB under high cholesterol. Increased ROS, BACE1, and Aβ levels by high cholesterol were decreased by NaB, but were not reduced by NaB and ATN-224 (Fig. 6e–h). To verify more clearly the effect of NaB on high cholesterol-induced ROS, BACE1, and Aβ levels through SOD1 expression, we performed *SOD1* siRNA transfection. Consistently, our data showed that increased ROS, BACE1, and Aβ levels by high cholesterol were reduced by NaB, but were not recovered by NaB and *SOD1* siRNA transfection [Supplementary Fig. S9].

## Discussion

This study demonstrates the antioxidant effect of NaB on high cholesterol-induced ROS in SK-N-MC cells, ultimately inhibiting BACE1-dependent amyloidogenesis caused by excessive ROS. Although the antioxidant effect of NaB is well known^8,47^, inhibitory effect of NaB on Aβ secretion through the antioxidant effect in neurons under high cholesterol has not been reported. The reason for targeting NaB as a potential therapeutic substance in AD was inspired by “gut–brain axis”. We focused on the microbiota composition in obese patients who are more likely to get AD, because butyrate-producing bacteria have been observed to decrease in the gut of obese patients. Therefore, we investigated the butyrate-producing bacteria that generally belong to Firmicutes at phylum level: in previous studies, the alteration of Firmicutes in the gut of obese patients or mice is controversial^48–50^. Our data showed that Firmicutes were hardly changed in HFD-fed mice, which indicates that different results appear to be dependent on age, sex, mouse types, and breeding conditions such as housing density, so it is necessary to conduct further investigations. Therefore, we collected plasma from mice and measured the concentration of NaB to see if there was actually less NaB from obese mice. Our results showed lower concentrations of NaB in obese mice plasma, which may suggest that cross of NaB into the blood brain barrier can be lower in obese patients. Furthermore, our data revealed that NaB reduced only BACE1 levels under high cholesterol, which seems to be dependent on cell or treated drug types. We also compared inhibitory effect of SCFAs on amyloidogenesis. Because NaP is also an HDAC inhibitor, and SCFAs affect cell signaling pathways as GPCR ligands^6,51^, it is necessary to confirm whether NaB has a stronger inhibitory effect on high cholesterol-induced Aβ secretion. In our results, only NaB was found to affect a reduction of BACE1 levels. Therefore, we hypothesized that NaB inhibits BACE1-dependent Aβ accumulation caused by high cholesterol.

NaB is absorbed in cells and acts as an HDAC inhibitor and also well known as a GPCR ligand, especially Gαi, influencing the cellular signal transduction. Actually, a number of studies reported diverse mechanisms of the antioxidant effect of NaB in various cell types^47,52,53^. Therefore, we investigated whether NaB acts as an HDAC inhibitor or a GPCR ligand to inhibit amyloidogenesis caused by high cholesterol. Our results suggest that NaB acts as an HDAC inhibitor, reducing high-cholesterol-induced amyloidogenesis. Although previous studies suggested that NaB has the strongest antioxidant effect among SCFAs, comparative experiment was performed by comparing the antioxidant effect of SCFAs [Supplementary Fig. S10]. There is no study on why they have same function but different effects, but this study will help to identify the mechanisms of inhibitory effect of NaB on Aβ secretion.

A number of studies described mtROS as major source of excessive ROS, when various cells including neuronal cells are exposed to lethal conditions such as high cholesterol or high glucose^54,55^. Recently, however, there is an increasing number of studies describing the major sources of pathological oxidative stress as NOX family in the CNS, such as microglia and astrocytes as well as neurons^20^. Thus, the major ROS sources appear to be dependent on neuronal cell types or concentrations, times, and types of treatment drug, so further studies are needed. The major ROS source of SK-N-MC cells in hypercholesterolemic environment is not known. Our results indicate that excessive ROS of cells caused by high cholesterol are mainly produced by NOX2. We thereby demonstrated that NaB prevents excessive ROS through reduction of NOX2 levels. In other words, the effect of NaB on neurons under high cholesterol is to suppress NOX2 levels, which leads to reduction of ROS formation.

It has been reported that the expression levels of antioxidant enzymes are increased as a protective effect against oxidative stress, whereas oxidative damage such as hyperglycemia inhibits NRF2 pathway, which makes cells vulnerable to oxidative stress^56^. Consistently, our results showed that NRF2 activity was reduced under high cholesterol, but NaB increased the nuclear translocation of NRF2. However, the specific mechanisms of antioxidant effect of NaB under high cholesterol are not known yet. In our results, p21 levels were decreased by high cholesterol and recovered by NaB, and p21/NRF2 colocalization was also increased, which leads to NRF2 stabilization. However, p21 is a typical cyclin-dependent kinase inhibitor, which is an apoptosis marker or apoptosis inhibition marker^57^. In addition, there are studies that NaB contributes to protecting neurons from cell death but adverse studies that NaB induces apoptosis in Caco-2^58,59^. Therefore, we demonstrated that increased cell death by high cholesterol was reduced by NaB, which means that increased p21 levels by NaB do not induce apoptosis [Supplementary Fig. S11]. Although previous studies reported that both p53 and Sp1 are critical transcription factors of p21^43^, our results showed that increased p21 levels by NaB are due to Sp1, not p53 [Supplementary Fig. S12].

NRF2 is a transcription factor that plays an anti-inflammatory role and main function of NF-κB is to regulate the inflammatory response^60,61^. As we referred to studies that NRF2 pathway inhibits NF-κB activation or an increase in NRF2 activity prevents NF-κB activation and even activation of NF-κB affects NRF2/Keap1/ARE signaling pathway^45,60,62^, we need to investigate the crosstalk between two transcription factors, especially under high cholesterol. Our results showed that *NRF2* knockdown enhanced NF-κB activation, followed by increased NOX2 levels. Therefore, considering the references and our results, we suggest that NRF2 stabilization by NaB suppresses NOX2 levels by weakening NF-κB activation. However, previous study reported that full transcriptional activity of NF-κB is regulated by acetylation at lysine 310^63^. Although NaB may directly affects NF-κB activation as an HDAC inhibitor, NaB seems to indirectly influence NF-κB activation via NRF2 stabilization, considering our results showing the correlation between NRF2 and NF-κB. However, the specific mechanism by which NaB modulates NF-κB activity needs further study.

In general, previous studies described regulation of transcription factors activation through phosphorylation of transcription factors^64,65^. However, the nuclear translocation of several transcription factors is known to be affected by acetylation, because the acetylation influences stability of transcription factors or protein–protein interaction^66^. However, the activity of transcription factors may be increased or decreased by acetylation, which depends on types of transcription factors or cells^44,67,68^. Although there is no report that regulation of Sp1 acetylation affects Sp1 activation, our data demonstrated that the acetylation and nuclear translocation of Sp1 decreased by high cholesterol were recovered by NaB. Thus, these findings suggested that NaB increases p21 levels by increasing nuclear translocation of Sp1 through regulation of Sp1 acetylation, and eventually induces NRF2 stabilization by contributing to p21/NRF2 colocalization, which is the correlation of NRF2, Sp1, and p21. Subsequently, our data showed that SOD1 of antioxidant enzymes was decreased under high cholesterol and recovered by NaB, but the others were not changed. Alteration of only SOD1 levels seems to be dependent on cell or treated drug types. Therefore, these results suggest that high cholesterol-induced ROS are eliminated by upregulating SOD1 levels via NRF2 stabilization by NaB, which is ultimately to prevent high cholesterol-induced amyloidogenesis.

**Fig. 7:**
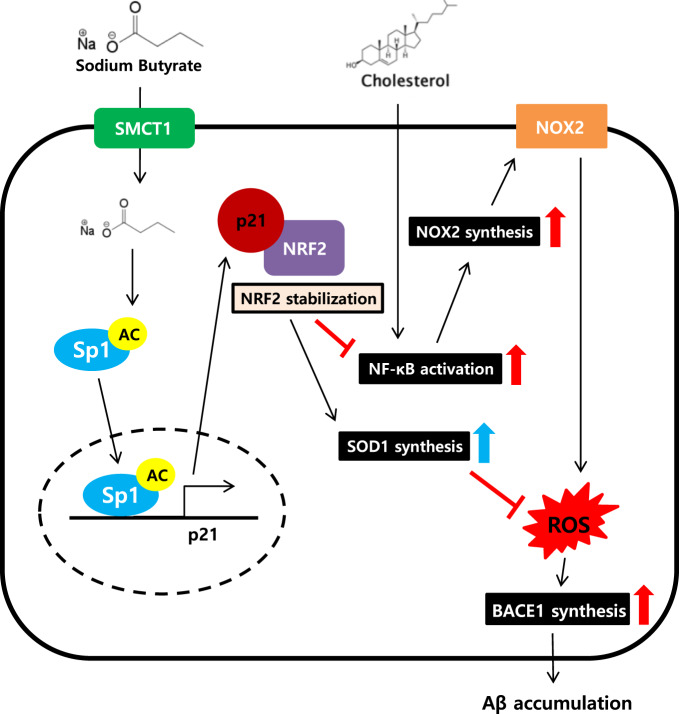
A hypothetical model for inhibition of high cholesterol-induced neuronal amyloidogenesis by the dual effects of NaB. NaB prevents high cholesterol-induced excessive ROS in SK-N-MC cells through the dual effects of NaB. NaB leads to NRF2 stabilization, followed by suppression of NOX2 levels via reducing the nuclear translocation of NF-κB and upregulation of SOD1 levels. NaB ultimately inhibits BACE1-dependent high cholesterol-induced neuronal amyloidogenesis by modulating NRF2 stabilization-mediated ROS levels.

Taken together, we first revealed the mechanisms of dual effects of NaB on neuronal cells under high cholesterol. Our study demonstrated that NaB protects SK-N-MC cells exposed to high cholesterol environment by modulating NRF2 stabilization-mediated ROS levels. In conclusion, we showed that NaB inhibits BACE1-dependent high cholesterol-induced neuronal amyloidogenesis by suppressing NOX2 levels and upregulating SOD1 levels via p21/NRF2 pathway (Fig. 7). Furthermore, it suggests that NaB can be a therapeutic strategic candidate to AD patients caused by obesity, because obese patients who are most likely to get AD showed reduced butyrate-producing bacteria in the microbiota composition.

## Materials and methods

### Materials

The human neuroblastoma cell line SK-N-MC was acquired from Korean Cell Line Bank (Seoul, Korea, RRID: CVCL_1398). Fetal bovine serum (FBS) and Dulbecco’s modified eagle medium (DMEM) were purchased from Hyclone (Logan, UT, USA). Antibiotics and serum replacement (SR) were acquired from Gibco (Grand Island, NY, USA). The antibodies of β-actin (sc-47778), Lamin A/C (sc-376248), GPX4 (sc-166570), catalase (sc-271803), NF-κB (sc-8008), BACE1 (sc-33711), and PSEN1 (sc-365450) were purchased from Santa Cruz Biotechnology (Santa Cruz, CA, USA). The antibodies of APP (ab32136), BACE1 (ab2077), Sp1 (ab227383), NRF2 (ab89443), and Aβ (ab2530) were purchased from Abcam (Cambridge, England). The antibody of NOX2 (611414) was purchased from BD bioscience. The antibody of SOD1 (CSB-PA02864A0Rb) was purchased from CusaBio (Houston, TX, USA) and SOD2 (06−984) was purchased from EMD Millipore (Burlington, MA, USA). The antibody of Sp1 (H00006667-MO3) was purchased from Novus (Littleton, CO, USA) and Acetylated-Lysine (9441 S) was purchased from Cell Signaling Technology (Danvers, MA, USA). The antibodies of p53 (PA5-27822) and p21 (MA5-14949) were purchased from Thermo Fisher and TurboFect, human or mouse Aβ (1–42) ELISA kit, CM-H2DCFDA, MitoSOX^TM^ Red, and RNAlater^TM^ solution were also obtained from Thermo Fisher (Waltham, MA, USA). ATN-224 was purchased from CSNpharm and Bay11-7082 was purchased from Calbiochem. Paraformaldehyde (PFA) was purchased from Lugen (Bucheon, Korea). EzSubcell™ subcellular fractionation kit was acquired from Atto (Tokyo, Japan). Sodium acetate was purchased from FUJIFILM Wako Pure Chemical Corporation. NAC, Vas2870, mitotempo, sodium butyrate, sodium propionate, butyric acid, acrylic acid, m-phosphoric acid, propyl formate, cholesterol-water soluble, DAPI, Triton X-100, ibuprofen, and PTX were purchased from Sigma Chemical Company (St. Louis, MO, USA). mRNA primers for *APP, BACE1, PSEN1, NOX1, NOX2, NOX3, NOX4, SOD1, SOD2, catalase, GPX4*, and *ACTB* were purchased from Cosmo Genetech (Seoul, Korea). *SMCT1, NRF2, BACE1, NOX2, SOD1, p65, Sp1, p21* siRNA were acquired from Bioneer (Daejeon, Korea). NT siRNA was purchased from Dharmacon (Lafayette, CO, USA).

### Culture of SK-N-MC cells

SK-N-MC cells were cultured without a feeder layer in high-glucose Dulbecco’s Modified Eagle Medium (DMEM), supplemented with 10% FBS and 1% antibiotic–antimycotic solution at 37 °C with 5% CO_2_. Cells were grown in 60, 100 mm diameter culture dishes, or 12-, 96-well plates in an incubator maintained at 37 °C with 5% CO_2_. When the cells reach approximately 60% confluency, the medium was replaced with DMEM supplemented with 1% antibiotic–antimycotic solution and 2% of SR 24 h before the experiments. Cells were maintained in 2% SR in DMEM containing 1% antibiotics and agents such as cholesterol, NaB, and ibuprofen.

### Preparation of brain tissue of the HFD-induced obese mouse model

To induce obesity through a diet, 6-week-old male ICR mice were fed either a regular chow diet or a HFD (Rodent Diet with 60 kcal% Fat and 90 g Added NaCl /4057 kcal, Research Diets Inc., New Brunswick, NJ, USA) for 14 weeks. Mice were stabilized in the new environment for 3 days before experimentation. Mice were granted *ad libitum* access to food and water. The room was maintained under standard environmental conditions (24 ± 2 °C, 12/12 h light/dark cycle with lights on at 07:00 a.m.) Mice were randomly assigned. The experiments were performed according to the “Guide for Animal Experiments” (Edited by the Korean Academy of Medical Sciences) and approved by the Institutional Animal Care and Use Committee (IACUC) of Seoul National University (SNU-180802–2). The brains were removed and post-fixed by 4% paraformaldehyde in 0.1 M PBS for 12 h. The brain tissues were cryo-protected by overnight infusion with 90 % sucrose, and 30 µm-thick coronal sections were serially cut using a cryostat (Leica, Deerfield, Germany). The sections were transferred to 6-well plates containing PBS for further processing.

### Metagenomic 16 S rRNA sequencing and data analysis

Mouse fecal samples were stored in RNAlater^TM^ solution at −80 °C until processing. DNA extractions for 16 S rRNA analysis were performed by using FastDNA^®^ SPIN Kit for Soil (MP Biomedicals, Solon, CA, USA) based on manufacturer’s recommendations. The bacterial 16 S rRNA V3-V4 region was amplified in accordance with the Illumina 16 S Metagenomic Sequencing Library Preparation guide (Illumina, CA, USA), using the primers with added adapter overhang sequences:^69^ forward primer: 5’-TCGTCGGCAGCGTCAGATGTGTATAAGAGACAGCCTACGGGNGGCWGCAG -3’, reverse primer: 5’- GTCTCGTGGGCTCGGAGATGTGTATAAGAGACAGGACTACHVGGGTATCTAATCC -3’. Also, the MiSeq platform (Illumina, CA, USA) with the 2 × 300 bp paired-end sequencing was used for amplicon sequencing through standard Illumina sequencing protocols. A quality control for the raw sequences of each sample was examined using FastQC^70^. Then, QIIME2 (v.2019.4)^71^, a standardized pipeline for 16 S rRNA sequencing data was processed. The q2-cutadapt plugin^72^ was used for removing the primer sequences and The DADA2 software package^73^ wrapped in QIIME2 was used to quality control including quality filtering, denoising, and used to feature table construction containing amplicon sequence variants (ASVs). After that, the q2-phylogeny plugin was used for generating the phylogenetic tree. Furthermore, alpha diversity (α-diversity) and beta diversity (β-diversity) were performed as the core-metrics-phylogenetic in the QIIME2 diversity plugin provides these two types of diversity analysis. For taxonomic annotation, the QIIME2 feature-classifier plugin (classify-sklearn) with a pretrained Naïve Bayes classifier on the GreenGene database (v.13_8 with 99% similarity operational taxonomic units (OTUs))^74^ was performed. Finally, the Statistical Analysis of Metagenomic Profiles (STAMP) software^75^ was used for comparing the taxonomic profiling analysis between groups.

### Preparation of plasma samples

All procedures for preparation of samples were performed following the previous report^76^. Hundred microliters of internal standard solution (150 µM acrylic acid, 1500 µM m-phosphoric acid) was added to 200 µl plasma. Samples were vortexed for 5 min followed by centrifugation (30 min, 14,916 rpm) and left to solidify the precipitate for 30 min at 4 °C. Hundred microliters of the clear supernatant was transferred into a new tube and 100 µl washed propyl formate was added. Samples were vortexed for 5 min followed by centrifugation (10 min, 14,916 rpm) before transferring 50 µl of the organic layer into GC vials (Waters, Milford, Massachusetts, United States) for analysis.

### GC–MS analysis

One microliter sample was injected into a straight glass liner, held at 200 °C. Helium (1 ml/min) was used as carrier gas in TR-FAME (25 m × 0.32 mm × 0.25 µm). The initial oven temperature of 60 °C was maintained for 4 min, then ramped to 130 °C at 50 °C/min and held for 3.7 min and finally raised to 240 °C at 30 °C/min and held for 10 min. The transfer line and ion source temperature is 250 °C. All samples, standards, and blanks were analyzed randomly at the GC–MS system. Trace 1300 Series gas chromatograph with TSQ Series mass spectrometer (Thermo Fisher, MA, USA) was used. Integrations were performed automatically with Triplus RSH autosampler (Thermo Fisher, MA, USA).

### Western blot analysis

Cells were detached from the 60 or 100 mm diameter culture dishes with a scraper and gathered by centrifugation (13,200 rpm, 4 °C, 5 min). Harvested cells and brain tissues were lysed by RIPA lysis buffer (ATTO Corporation, Tokyo, Japan) and incubated for 30 min on ice. The lysates were then cleared by centrifugation (13,200 rpm, 4 °C, 30 min). The protein concentration was measured by the bicinchoninic acid (BCA) assay kit (Bio-Rad, Hercules, CA, USA). Samples containing 10 µg of protein were prepared for 8–15% sodium dodecyl sulfate-polyacrylamide gel electrophoresis (SDS-PAGE) and then transferred to a polyvinylidene fluoride (PVDF) membrane and blocked with 5% skim milk (Gibco) dissolved in TBST for 40 min. The blocked membranes were washed with TBST and incubated with primary antibody overnight at 4 °C. The membranes were subsequently washed and incubated with HRP-conjugated secondary antibody at room temperature for 2 h. The western blotting bands were visualized by means of chemiluminescence (Bio-Rad, Hercules, CA, USA). Specific bands were detected using a ChemiDoc™ XRS+System (Bio-Rad, Hercules, CA, USA) and analyzed by using ImageJ software.

### Reverse transcription-polymerase chain reaction (RT-PCR) and real-time PCR

RNA was extracted using MiniBEST Universal RNA Extraction Kit (TaKaRa, Otsu, Shinga, Japan). Reverse transcription was performed using 1 µg of RNA with a Maxime RT-PCR PreMix Kit (Intron Biotechnology, Seongnam, Korea) to obtain cDNA. Two microliters of cDNA were then amplified using Quanti NOVA SYBR Green PCR Kits (Qiagen, Hilden, Germany). Real-time quantification of RNA targets was performed in a Rotor-Gene 6000 real-time thermal cycling system (Corbett Research, NSW, Australia). The reaction mixture (20 µl) contained 200 ng of total RNA, 0.5 mM of each primer for the gene of interest, and appropriate amounts of enzymes and fluorescent dyes as recommended by the manufacturer. The real-time PCR was performed as follows: 15 min at 95 °C for DNA polymerase activation; 15 s at 95 °C for denaturing; and 50 cycles of 15 s at 94 °C, 30 s at 54 °C, and 30 s at 72 °C. Data were collected during the extension step, and analysis was performed using the software provided; melting curve analysis was performed to verify the specificity and identity of the PCR products. Normalization of gene expression levels was performed by using the *ACTB* gene as an endogenous control. Sequences of the primers used are described in Supplementary Table S2.

### Detection of intracellular ROS and mitochondrial ROS

The CM-H2DCFDA and MitoSOX^TM^ Red were used to measure the intracellular ROS and mitochondrial ROS respectively. Cells were seeded in a 96-well plate at same density (2 × 10^3^ cells per well). Cells were treated with 5 µM DCF-DA or 2 µM MitoSOX^TM^ Red and then incubated in an incubator at 37 °C with 5% CO_2_ for 30 min or 15 min, respectively. Cells were washed twice with PBS and then measured with a luminometer (Victor3; PerkinElmer Inc., Waltham, MA, USA). The fluorescence intensity of CM-H2DCFDA was measured with a luminometer at an excitation and emission wavelength of 485 and 535 nm and MitoSOX^TM^ Red was measured at an excitation and emission wavelength of 530 and 580 nm.

### Flow cytometry

Cells were seeded in 12-well culture dishes. When cells were at 60% confluence, medium was replaced with 2% SR in DMEM containing 1% antibiotics for 24 h. Cells were stained with 5 µM DCF-DA for 30 min or 2 µM MitoSox^TM^ Red for 15 min. Cells were washed with PBS two times and treated with a 0.05% trypsin and 0.5 mM EDTA solution to detach cells from the dish for 3 min. Cells were centrifuged at 3000 rpm for 5 min. DCF-DA or MitoSOX^TM^ Red staining were determined via flow cytometry (CytoFlex; Beckman Coulter, Fullerton, CA, USA).

### Nuclear fractionation

To prepare the cytosolic and nuclear-fractionized samples, the EzSubcell™ subcellular fractionation/extraction kit was used. All procedures of the subcellular fractionation assay followed manufacturer’s protocol. Nuclear NF-κB, NRF2, p53, and Sp1 expression levels in nuclear-fractionized samples were normalized by Lamin A/C expression levels.

### siRNA transfection

When cells were grown until 60% confluence, cells were transfected with *SMCT1, NRF2, BACE1, NOX2, SOD1, p65, Sp1, p21* siRNA, and nontargeting (NT) siRNA for 12 h using TurboFectTM transfection reagent in 2% SR in DMEM containing 1% antibiotics. After 12 h of incubation, the culture media were replaced with 2% SR in DMEM containing 1% antibiotics and the cells were maintained for 24 h. The concentration of each transfected siRNA was 25 nM. The sequences of siRNAs used are described in Supplementary Table S3.

### Immunocytochemistry

Cells were washed with PBS on a confocal dish. Cells were fixed with 4% paraformaldehyde (PFA) for 10 min and then, incubated in 0.1% Triton X-100 for membrane permeabilization for 10 min. Cells were incubated with 5% normal goat serum in PBS for 30 min and with primary antibodies overnight at 4 °C. Next, Cells were washed with PBS three times and incubated with Alexa Fluor™ 488 or 555-conjugated secondary antibodies for 2 h at room temperature. Immunofluorescence images were obtained by a super-resolution radial fluctuations (SRRF) imaging system (Andor Technology, Belfast, UK)^77^.

### Immunohistochemistry

Tissues were deparaffinized with xylene and 100, 95, 70, and 50% ethanol. For peroxide inactivation, the tissues were then incubated on 3% H_2_O_2_ in methanol and washed with PBS. For permeabilization, the tissues were incubated in PBS containing 0.5% Triton x100 for 15 min, washed with PBS, and incubated with 5% NGS in PBS for 30 min. The tissues were labeled with 5% NGS in PBS containing APP, BACE1, and Aβ antibodies at a ratio of 1:100 for 2 h, followed by secondary antibody and PI in 1:100 ratio for 1 h at room temperature. After washing with PBS for 15 min, images were obtained by using FluoView^TM^ 300 confocal microscope (Olympus).

### Aβ ELISA

The Aβ (1–42) concentration level in medium sample was measured by commercial enzyme-linked immunosorbent assay (ELISA) kits. Medium samples were collected and centrifugated at 13,200 rpm for 5 min and the supernatant samples were collected as ELISA samples. All procedures of the Aβ ELISA assay were performed according to the manufacturer’s protocol.

### Water soluble tetrazolium salt (WST-1) assay

WST-1 assay was used for determining cell proliferation and cell viability in vitro model. All procedures of the assay were conducted according to the manufacturer’s protocol. Cells were seeded in 96-well plate (2 × 10^3^ cells per well). EZ-Cytox^TM^ reagent was added to each well and the cells were incubated for 1 h in 5% CO_2_ incubator at 37 °C. The absorbance of each sample was measured at a wavelength of 450 nm by using a microplate reader.

### Trypan-blue cell viability assay

The SK-N-MC cells were washed with PBS, and then incubate with a 0.05% trypsin and 0.5 mM EDTA solution to detach the cells. Trypsin inhibitor (PBS with 10% FBS) was added to quench trypsin. The cell suspension solution was centrifugated 3,000 rpm for 5 min. The cell pellet was suspended with 0.4% trypan blue in PBS to stain the dead cells. Ten microliters was dispensed on the cell counting chamber slides. Trypan-blue stained or unstained cells were counted by using a Countess II FL (Thermo Fisher, MA, USA).

### Statistical analysis

For the reliability of the experiment, we randomized through each experiment and performed the experiments. All samples were randomly assigned following simple randomization procedures (computerized random numbers) to one of two treatment groups. All experiments in vitro and animal tests were performed and assessed in a blinded-fashion. The number of replicates was 3–4 throughout all experiments. We used SigmaPlot (version 12.0, Systat Software Inc.) to choose the appropriate sample size: The sample size is calculated considering the power (1-beta) for statistical validity and Difference in Means, Standard Deviation of the expected result. We established the inclusion/exclusion criteria to derive more accurate and reliable data. Because some outliers may have significant something, we valued to meet the pretest data. Therefore, we excluded the specific confounding variables compared with pretest data. Quantitative data are presented as the mean ± standard error of the mean (S.E.M). Statistical analysis was conducted by using GraphPad Prism Version 6.0 (GraphPad Inc., San Diego, CA, USA). All data achieved normality and were analyzed by parametric statistics through the GraphPad Prism Version 6.0. The variation estimate of the data acquired through the pretest was obtained, and then it was confirmed that the normal distribution was achieved. Based on this, the experiments were performed, and the data actually satisfied the normality. Statistical differences between more than two groups and within groups variance were assessed by using analysis of variance (ANOVA). One-way ANOVA (with Dunnett’s multiple comparison test) or Two-way ANOVA (with Tukey’s multiple comparison test) were used for analyzing the differences among groups. The student’s *t*-test was conducted for comparing the means of treatment groups with that of the control group. *p* < 0.05 was considered statistically significant.

## Supplementary information

Supplementary tables

Supplementary figure legends-no coloured

Supplementary figure S1

Supplementary figure S2

Supplementary figure S3

Supplementary figure S4

Supplementary figure S5

Supplementary figure S6

Supplementary figure S7

Supplementary figure S8

Supplementary figure S9

Supplementary figure S10

Supplementary figure S11

Supplementary figure S12
