# Supplementary material for: Sodium butyrate inhibits high cholesterol-induced neuronal amyloidogenesis by modulating NRF2 stabilization-mediated ROS levels: involvement of NOX2 and SOD1
Source: Cell Death Dis. 2020 Jun 18;11(6):469. doi: 10.1038/s41419-020-2663-1 (PMC7303181; doi:10.1038/s41419-020-2663-1)
Supplement: Supplementary file 1 — Supplementary tables [file 41419_2020_2663_MOESM1_ESM.docx]

**Table S1. List of *p*-value in phylum, order, and family level of gut microbiota in the obesity model**

**Phylum**

| Feature | Diff. between means | *p*-value |
| --- | --- | --- |
| p__Bacteroidetes | 7.260 | 0.041 |
| p__Verrucomicrobia | -6.134 | 0.100 |
| p__Deferribacteres | -0.205 | 0.193 |
| p__Tenericutes | -0.284 | 0.287 |
| p__Proteobacteria | 2.282 | 0.318 |
| p__TM7 | -0.022 | 0.343 |
| p__Actinobacteria | -0.432 | 0.450 |
| p__Firmicutes | -2.473 | 0.577 |
| Unclassified k__Bacteria | 0.007 | 0.616 |

**Order**

| Feature | Diff. between means | *p*-value |
| --- | --- | --- |
| o__Turicibacterales | 1.576 | < 0.01 |
| o__SHA-98 | 0.025 | < 0.01 |
| o__Coriobacteriales | -0.937 | < 0.01 |
| o__Erysipelotrichales | -1.749 | < 0.01 |
| o__Bacteroidales | 7.260 | 0.041 |
| o__RF32 | 4.000 | 0.076 |
| o__Verruoomicrobiales | -6.134 | 0.100 |
| o__Bacillales | -0.083 | 0.141 |
| o__Burkholderialed | -1.821 | 0.148 |
| o__Deerribacterales | -0.205 | 0.193 |
| o__Bifidobactetiales | -0.505 | 0.256 |
| o__RF39 | -0.035 | 0.298 |
| o__Anaeroplasmatales | -0.249 | 0.309 |
| o__I025 | -0.022 | 0.343 |
| o__Lactobacillales | -2.467 | 0.379 |
| Unclassified k_Bacteria | 0.007 | 0.616 |
| o__Enterobacteriales | 0.103 | 0.776 |
| o__Clostridiales | 0.226 | 0.951 |

**Family**

| Feature | Diff. between means | *p*-value |
| --- | --- | --- |
| f__Peptococcaceae | -2.063 | < 0.01 |
| f__Peptostreptococcaceae | -2.939 | < 0.01 |
| f__Turicibacteraceae | 1.576 | < 0.01 |
| Unclassified o__SHA-98 | 0.025 | < 0.01 |
| f__Ruminococcaceae | 4.398 | < 0.01 |
| f__Coriobacteriaceae | -0.937 | < 0.01 |
| f__S24-7 | 2.525 | < 0.01 |
| f__Streptococcaceae | -0.122 | < 0.01 |
| f__Erysipelotrichaceae | -1.749 | < 0.01 |
| f__Porphyromonadaceae | -3.452 | < 0.01 |
| f__Bacteroidaceae | 8.187 | 0.011 |
| f__Clostridiaceae | -0.703 | 0.040 |
| f__Enterococcaceae | -0.392 | 0.042 |
| Unclassified o__RF32 | 4.000 | 0.076 |
| f__Lachnospiraceae | 2.799 | 0.094 |
| f__Dehalobacteriaceae | -0.022 | 0.099 |
| f__Verrucomicrobiaceae | -6.134 | 0.100 |
| f__Christensenellaceae | -0.030 | 0.124 |
| f__Staphylococcaceae | -0.083 | 0.141 |
| f__Alcaligenaceae | -1.821 | 0.148 |
| f__Deferribacteraceae | -0.205 | 0.193 |
| f__Bifidobacteriaceae | 0.505 | 0.256 |
| Unclassified o__RF39 | -0.035 | 0.298 |
| f__Anaeroplasmataceae | -0.249 | 0.309 |
| f__Rs-045 | -0.022 | 0.343 |
| Unclassified o__Lactobacilliales | -0.016 | 0.343 |
| f__Lactobacilliaceae | -1.937 | 0.480 |
| f__[Mogibacteriaceae] | 0.059 | 0.544 |
| f__Veilonellaceae | -0.047 | 0.579 |
| Unclassified o__Clostridiales | -1.226 | 0.595 |
| Unclassified k__Bacteria | 0.007 | 0.616 |
| f__Enterobacteriaceae | 0.103 | 0.776 |

**Table S2. Sequences of primers used for RT-PCR and real-time PCR**

| Gene | Identification | Sequence (5'-3') |
| --- | --- | --- |
| *APP* | Forward | CTGGCTGAAGAAAGTGACAATG |
|  | Reverse | TCCTCTACCTCATCACCATCCT |
| *BACE1* | Forward | GGAGTACAAAGACAGGGAATAG |
|  | Reverse | GAATAAAGGGTGGTTCAGATAGA |
| *PSEN1* | Forward | AGGAAAGGGGAGTAAAACTTGG |
|  | Reverse | AAAGGTGATGGAGATTGGAAGA |
| *NOX1* | Forward | TCCCAGCAGAAGGTTGTGATTACC |
|  | Reverse | TGCCATTCCAGGAGAGAGATTGAG |
| *NOX2* | Forward | CTGCTCAACAAGAGTTCGAAGA |
|  | Reverse | GCCTCCTTCAGGGTTCTTTATT |
| *NOX3* | Forward | ACAACATCACCTTCTGTAGAGACCG |
|  | Reverse | AATCCATTTCCAAGCCGAGG |
| *NOX4* | Forward | GACTTTACAGGTATATCCGGAGCAA |
|  | Reverse | TGCAGATACACTGGACAATGTAGA |
| *SOD1* | Forward | CAATTTCGAGCAGAAGGAAAGT |
|  | Reverse | ACCGTGTTTTCTGGATAGAGGA |
| *SOD2* | Forward | ATGGTGGTGGTCATATCAATCA |
|  | Reverse | TGTAAGTGTCCCCGTTCCTTAT |
| *Catalase* | Forward | GACCGAGAGAGAATTCCTGAGA |
|  | Reverse | CGAGATCCCAGTTACCATCTTC |
| *GPX4* | Forward | CATGGTTAACCTGGACAAGTACC |
|  | Reverse | GCAGATCTTGCTGAACATATCG |
| *ACTB* | Forward | AACCGCGAGAAGATGACC |
|  | Reverse | AGCAGCCGTGGCCATCTC |

**Table S3. Sequences of siRNAs used for gene silencing**

| Target gene | Sequence | Supplier |
| --- | --- | --- |
| *SMCT1* | GAAUUCUGGCAUUGACAUU  AAUGUCAAUGCCAGAAUUC | Bioneer |
|  | CUGGAUUUGCAUCCGUGAU  AUCACGGAUGCAAAUCCAG |  |
|  | CAGUUAUCUGGACAGAUGU  ACAUCUGUCCAGAUAACUG |  |
| *NRF2* | GAGACUACCAUGGUUCCAA  UUGGAACCAUGGUAGUCUC | Bioneer |
|  | CAGCUAUGGAGACACACUA  UAGUGUGUCUCCAUAGCUG |  |
|  | GACAAGCUGGUUGAGACUA  UAGUCUCAACCAGCUUGUC |  |
| *BACE1* | CUCUGAAGAGCUGAGUACU  AGUACUCAGCUCUUCAGAG | Bioneer |
|  | GUCCUAGUUUUAGACCUCA  UGAGGUCUAAAACUAGGAC |  |
|  | CUGUUCAUGACAGCUACUA  UAGUAGCUGUCAUGAACAG |  |
| *NOX2* | GUAAUGUCAGUGGAAGUUA  UAACUUCCACUGACAUUAC | Bioneer |
|  | CUGAGUAAACAAAGCAUCU  AGAUGCUUUCUUUACUCAG |  |
|  | GAGCAUACUCCAGUUUACU  AGUAAACUGGAGUAUGCUC |  |

| *SOD1* | GAAAACACGGUGGGCCAAA  UUUGGCCCACCGUGUUUUC | Bioneer |
| --- | --- | --- |
|  | GGUGGUCCAUGAAAAAGCA  UGCUUUUUCAUGGACCACC |  |
|  | GACUUGGGCAAAGGUGGAA  UUCCACCUUUGCCCAAGUC |  |
| *p65* | CGAGUGAACCGAAACUCUG  CAGAGUUUCGGUUCACUCG | Bioneer |
|  | GGUGUAUUUCACGGGACCA  UGGUCCCGUGAAAUACACC |  |
|  | CUCUUCUCAAGUGCCUUAA  UUAAGGCACUUGAGAAGAG |  |
| *Sp1* | CUCUCAGGACAGACUCAGU  ACUGAGUCUGUCCUGAGAG | Bioneer |
|  | CGUAGUCAGCUUCAGGAGU  ACUCCUGAACCUGACUACG |  |
|  | GAGUCAGGAGCUUAUUACA  UGUAAUAAGCUCCUGACUC |  |
| *p21* | CUGUACUGUUCUGUGUCUU  AAGACACAGAACAGUACAG | Bioneer |
|  | CAGUUCAUUGCACUUUGAU  AUCAAAGUGCAAUGAACUG |  |
|  | CUCAUCCCGUGUUCUCCUU  AAGGAGAACACGGGAUGAG |  |
| Non-targeting (NT) | UAGCGACUAAACACAUCAA | Dharmacon |
|  | UAAGGCUAUGAAGAGAUAC |  |
|  | AUGUAUUGGCCUGUAUUAG |  |
|  | AUGAACGUGAAUUGCUCAA |  |
