# Supplementary material for: Sodium butyrate inhibits high cholesterol-induced neuronal amyloidogenesis by modulating NRF2 stabilization-mediated ROS levels: involvement of NOX2 and SOD1
Source: Cell Death Dis. 2020 Jun 18;11(6):469. doi: 10.1038/s41419-020-2663-1 (PMC7303181; doi:10.1038/s41419-020-2663-1)
Supplement: Supplementary file 2 — Supplementary figure legends-no coloured [file 41419_2020_2663_MOESM2_ESM.docx]

**Title**

Sodium butyrate inhibits high cholesterol-induced neuronal amyloidogenesis by modulating NRF2 stabilization-mediated ROS levels: Involvement of NOX2 and SOD1

**Authors**

Seo Yihl Kim^1^, Chang Woo Chae^1^, Hyun Jik Lee^4,5^, Young Hyun Jung^1^, Gee Euhn Choi^1^, Jun Sung Kim^1^, Jae Ryong Lim^1^, Joo Eun Lee^1^, Ji Hyeon Cho^1^, Hansoo Park ^2,3^, Changho Park ^3^ and Ho Jae Han^1, *^

**Affiliations**

^1^Department of Veterinary Physiology, College of Veterinary Medicine, Research Institute for Veterinary Science, and BK21 PLUS Program for Creative Veterinary Science Research, Seoul National University, Seoul 08826, Republic of Korea.

^2^Department of Biomedical Science and Engineering, Gwangju Institute of Science and Technology (GIST), Gwangju, Korea.

^3^Genome and Company, Pangyo-ro 253, Bundang-gu. Seoungnam-si, Gyeonggi-do 13486, Korea.

^4^Laboratory of Veterinary Physiology, College of Veterinary Medicine, Chungbuk National University, Cheongju, Chungbuk 28644, South Korea.

^5^Institute for Stem Cell & Regenerative Medicine (ISCRM), Chungbuk National University, Cheongju, 28644, Chungbuk, Korea.

^*^Corresponding author**:** Ho Jae Han, D.V. M, Ph.D

Professor of Department of Veterinary Physiology

College of Veterinary Medicine, Seoul National University, Seoul 08826, Republic of Korea

Tel: +82-2-880-1261

E-mail address: [hjhan@snu.ac.kr](mailto:hjhan@snu.ac.kr)

Tel: +82-2-880-1261

Fax: +82-2-880-2732

Running title: Effect of sodium butyrate on neuronal cells

**Supplementary figure S1. Alteration of total cholesterol levels in plasma of obesity model.** Blood samples were taken from the mice, and then red blood cells were removed by centrifugation for 5 min at 3,000 rpm for plasma preparation. The total cholesterol levels were measured by serum biochemistry instrument (Hitachi 7180, Fuji dry type, IDEXX Catalyst, Easy-Lyte Plus electrolyte). Data are presented as a mean ± S.E.M. *n* = 4. **p* < 0.05 versus ND.

**Supplementary figure S2. Involvement of BACE1 in Aβ accumulation under high cholesterol conditions.** SK-N-MC cells were transfected with *BACE1* siRNA for 12 h. Cells were treated with high cholesterol for 72 h. Aβ concentration of medium samples was detected by using ELISA kit. Data are presented as a mean ± S.E.M. *n* = 4. ^*^*p* < 0.05 versus NT siRNA transfection, ^#^*p* < 0.05 versus high cholesterol treatment with NT siRNA transfection. Data are presented as a mean ± S.E.M. *n* = 3. ^*^*p* < 0.05 versus control, ^#^*p* < 0.05 versus high cholesterol treatment.

**Supplementary figure S3. Gradient PCR of NOX isotypes for real-time PCR.** SK-N-MC cells were pre-treated with NaB for 30 min prior to high cholesterol treatment for 24 h. Gradient PCR was performed with complementary DNA of SK-N-MC cells. 1: 52 °C, 2: 55 °C, 3: 58 °C. Optimum annealing temperature was set at 58 °C.

**Supplementary figure S4. Involvement of NF-κB in high cholesterol-induced ROS generation, NOX2 and BACE1 expression, and Aβ accumulation. A** SK-N-MC cells were pre-treated with Bay11-7082 (5 µM) for 30 min prior to high cholesterol treatment for 72 h where ROS with DCF-DA were measured by flowcytometer. Total cell counts = 1.0 × 10^4^ cells. Data are presented as a mean ± S.E.M. *n* = 3. ^*^*p* < 0.05 versus control, ^#^*p* < 0.05 versus high cholesterol treatment. **B** Cells were transfected with *p65* siRNA or NT siRNA for 12 h, and treated with high cholesterol for 72 h where ROS with DCF-DA were measured by flowcytometer. Total cell counts = 1.0 × 10^4^ cells. Data are presented as a mean ± S.E.M. *n* = 3. **C-D** Cells were transfected with *p65* siRNA or NT siRNA for 12 h, and treated with high cholesterol for 24 h. The expression levels of NOX2 and BACE1 were analyzed by western blot. β-actin was used as a loading control. Data are presented as a mean ± S.E.M. *n* = 4. **E** Cells were transfected with *p65* siRNA or NT siRNA for 12 h, and treated with high cholesterol for 72 h. Aβ concentration of medium samples was detected by using ELISA kit. Data are presented as a mean ± S.E.M. *n* = 4. ^*^*p* < 0.05 versus NT siRNA transfection, ^#^*p* < 0.05 versus high cholesterol treatment with NT siRNA transfection. All blot images shown are representative.

**Supplementary figure S5.** **Involvement of NOX2 in high cholesterol-induced BACE1 expression and Aβ accumulation. A** SK-N-MC cells were transfected with *NOX2* siRNA for 12 h. Cells were treated with high cholesterol for 24 h. The expression levels of BACE1 were analyzed by western blot. β-actin was used as a loading control. Data are presented as a mean ± S.E.M. *n* = 4. **B** Cells were transfected with *NOX2* siRNA for 12 h and treated with high cholesterol for 72 h. Aβ concentration of medium samples was detected by using ELISA kit. Data are presented as a mean ± S.E.M. *n* = 4. ^*^*p* < 0.05 versus NT siRNA transfection, ^#^*p* < 0.05 versus high cholesterol treatment with NT siRNA transfection. All blot images shown are representative.

**Supplementary figure S6. Effect of NaB on high cholesterol-induced ROS generation, BACE1 expression and Aβ accumulation through NRF2. A** SK-N-MC cells were transfected with *NRF2* siRNA or NT siRNA for 12 h, and pre-treated with NaB for 30 min prior to treatment of high cholesterol for 72 h where ROS with DCF-DA were measured by flowcytometer. Total cell counts = 1.0 × 10^4^ cells. Data are presented as a mean ± S.E.M. *n* = 3. **B** Cells were transfected with *NRF2* siRNA or NT siRNA for 12 h, and pre-treated with NaB for 30 min prior to treatment of high cholesterol for 24 h. The expression levels of BACE1 were analyzed by western blot. β-actin was used as a loading control. Data are presented as a mean ± S.E.M. *n* = 4. **C** Cells were transfected with *NRF2* siRNA or NT siRNA for 12 h, and pre-treated with NaB for 30 min prior to treatment of high cholesterol for 72 h. Aβ concentration of medium samples was detected by using ELISA kit. Data are presented as a mean ± S.E.M. *n* = 4. ^*^*p* < 0.05 versus NT siRNA transfection, ^#^*p* < 0.05 versus high cholesterol treatment with NT siRNA transfection. ^$^*p* < 0.05 versus treatment of high cholesterol and NaB with NT siRNA transfection. All blot images shown are representative

**Supplementary figure S7. Effect of NaB on high cholesterol-induced ROS generation, BACE1 expression and Aβ accumulation through sp1. A** SK-N-MC cells were transfected with *sp1* siRNA or NT siRNA for 12 h, and pre-treated with NaB for 30 min prior to treatment of high cholesterol for 72 h where ROS with DCF-DA were measured by flowcytometer. Total cell counts = 1.0 × 10^4^ cells. Data are presented as a mean ± S.E.M. *n* = 3. **B** Cells were transfected with *sp1* siRNA or NT siRNA for 12 h, and pre-treated with NaB for 30 min prior to treatment of high cholesterol for 24 h. The expression levels of BACE1 were analyzed by western blot. β-actin was used as a loading control. Data are presented as a mean ± S.E.M. *n* = 4. **C** Cells were transfected with *sp1* siRNA or NT siRNA for 12 h, and pre-treated with NaB for 30 min prior to treatment of high cholesterol for 72 h. Aβ concentration of medium samples was detected by using ELISA kit. Data are presented as a mean ± S.E.M. *n* = 4. ^*^*p* < 0.05 versus NT siRNA transfection, ^#^*p* < 0.05 versus high cholesterol treatment with NT siRNA transfection. ^$^*p* < 0.05 versus treatment of high cholesterol and NaB with NT siRNA transfection. All blot images shown are representative.

**Supplementary figure S8. Effect of NaB on high cholesterol-induced ROS generation, BACE1 expression and Aβ accumulation through p21. A** SK-N-MC cells were transfected with *p21* siRNA or NT siRNA for 12 h, and pre-treated with NaB for 30 min prior to treatment of high cholesterol for 72 h where ROS with DCF-DA were measured by flowcytometer. Total cell counts = 1.0 × 10^4^ cells. Data are presented as a mean ± S.E.M. *n* = 3. **B** Cells were transfected with *p21* siRNA or NT siRNA for 12 h, and pre-treated with NaB for 30 min prior to treatment of high cholesterol for 24 h. The expression levels of BACE1 were analyzed by western blot. β-actin was used as a loading control. Data are presented as a mean ± S.E.M. *n* = 4. **C** Cells were transfected with *p21* siRNA or NT siRNA for 12 h, and pre-treated with NaB for 30 min prior to treatment of high cholesterol for 72 h. Aβ concentration of medium samples was detected by using ELISA kit. Data are presented as a mean ± S.E.M. *n* = 4. ^*^*p* < 0.05 versus NT siRNA transfection, ^#^*p* < 0.05 versus high cholesterol treatment with NT siRNA transfection. ^$^*p* < 0.05 versus treatment of high cholesterol and NaB with NT siRNA transfection. All blot images shown are representative.

**Supplementary figure S9. Effect of NaB on high cholesterol-induced ROS accumulation, BACE1 expression, and Aβ accumulation through SOD1 expression. A** SK-N-MC cells were transfected with *SOD1* siRNA or NT siRNA for 12 h, and pre-treated with NaB for 30 min prior to treatment of high cholesterol for 72 h where ROS with DCF-DA were measured by flowcytometer. Total cell counts = 1.0 × 10^4^ cells. Data are presented as a mean ± S.E.M. *n* = 3. **B** Cells were transfected with *SOD1* siRNA or NT siRNA for 12 h, and pre-treated with NaB for 30 min prior to treatment of high cholesterol for 24 h. The expression levels of BACE1 were analyzed by western blot. β-actin was used as a loading control. Data are presented as a mean ± S.E.M. *n* = 4. **C** Cells were transfected with *SOD1* siRNA or NT siRNA for 12 h, and pre-treated with NaB for 30 min prior to treatment of high cholesterol for 72 h. Aβ concentration of medium samples was detected by using ELISA kit. Data are presented as a mean ± S.E.M. *n* = 4. ^*^*p* < 0.05 versus NT siRNA transfection, ^#^*p* < 0.05 versus high cholesterol treatment with NT siRNA transfection. ^$^*p* < 0.05 versus treatment of high cholesterol and NaB with NT siRNA transfection. All blot images shown are representative.

**Supplementary figure S10. Effect of SCFAs on high cholesterol-induced ROS generation.** SK-N-MC cells were pre-treated with NaB or NaP (500 µM) or NaA (500 µM) for 30 min prior to high cholesterol treatment for 72 h where ROS with DCF-DA were measured by flowcytometer. Total cell counts = 1.0 × 10^4^ cells. Data are presented as a mean ± S.E.M. *n* = 4. ^*^*p* < 0.05 versus control, ^#^*p* < 0.05 versus high cholesterol treatment.

**Supplementary figure S11. Effect of NaB on apoptosis of SK-N-MC cells under high cholesterol conditions. A** SK-N-MC cells were pre-treated with NaB for 30 min prior to high cholesterol treatment for 48 h. Trypan blue stained or unstained cells were counted by using the Countess. Data are presented as a mean ± S.E.M. *n* = 4. ^*^*p* < 0.05 versus control, ^#^*p* < 0.05 versus high cholesterol treatment. **B** Cells were pre-treated with NaB for 30 min prior to high cholesterol treatment for 72 h. The absorbance of each sample was measured by using a microplate reader. Cell viability of SK-N-MC cells was measured by WST-1 assay. Data are presented as a mean ± S.E.M. *n* = 5. ^*^*p* < 0.05 versus control, ^#^*p* < 0.05 versus high cholesterol treatment.

**Supplementary figure S12. Effect of NaB on the nuclear translocation of p53 under high cholesterol conditions.** SK-N-MC cells were pre-treated with NaB for 30 min prior to high cholesterol treatment for 24 h. p53, Lamin A/C, and β-actin protein levels in cytosolic and nuclear-fractionized samples were analyzed by western blot. Data are presented as a mean ± S.E.M. *n* = 3. ^*^*p* < 0.05 versus control. ^#^*p* < 0.05 versus high cholesterol treatment. All blot images shown are representative.

**Table S1. List of *p*-value in phylum, order, and family level of gut microbiota in the obesity model**

**Table S2. Sequences of primers used for RT-PCR and real-time PCR**

**Table S3. Sequences of siRNAs used for gene silencing**
