# Supplementary figures and images for: Sodium butyrate inhibits high cholesterol-induced neuronal amyloidogenesis by modulating NRF2 stabilization-mediated ROS levels: involvement of NOX2 and SOD1
Source: Cell Death Dis. 2020 Jun 18;11(6):469. doi: 10.1038/s41419-020-2663-1 (PMC7303181; doi:10.1038/s41419-020-2663-1)

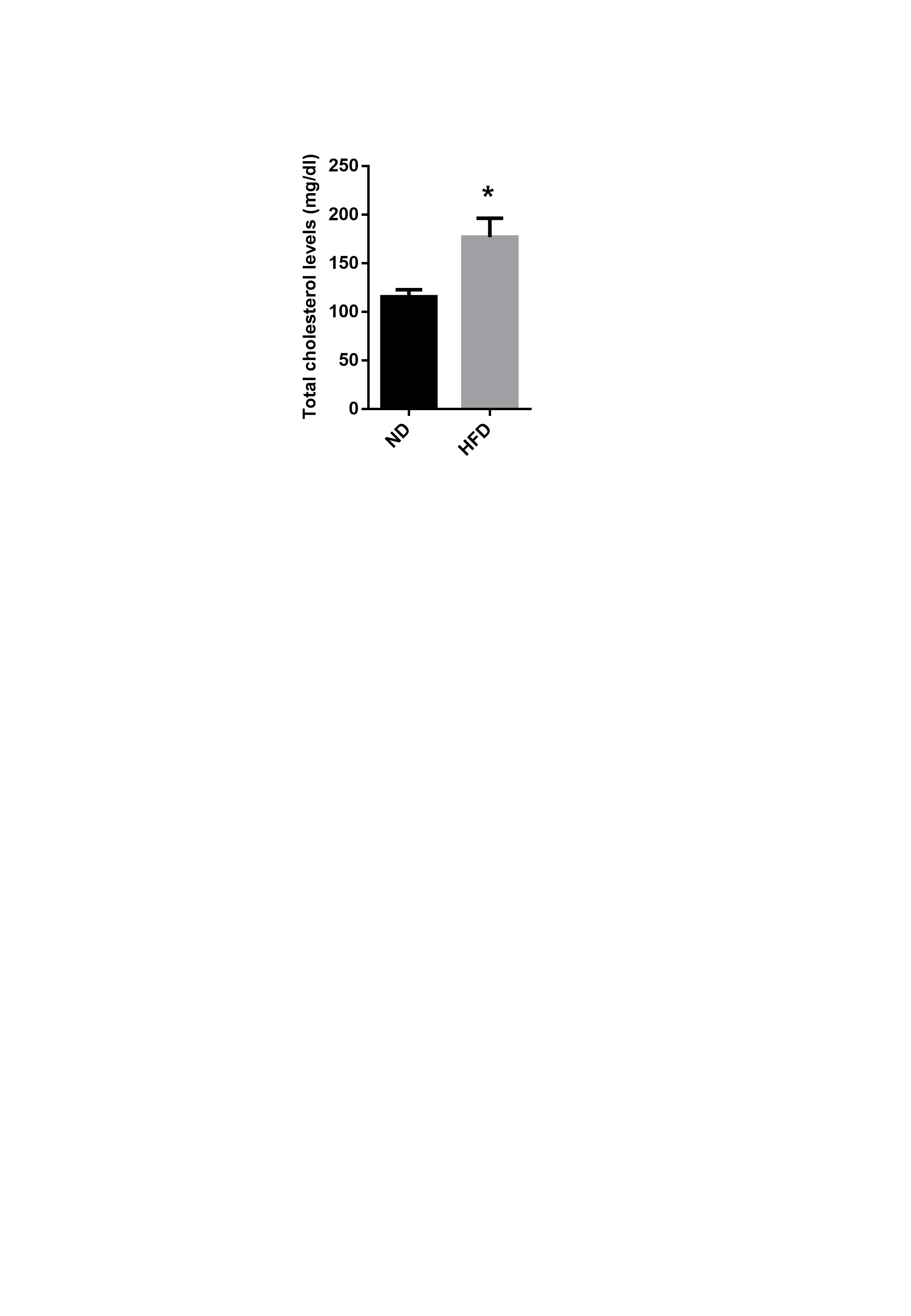

Supplement: Supplementary file 3 — Supplementary figure S1 [file 41419_2020_2663_MOESM3_ESM.tif]

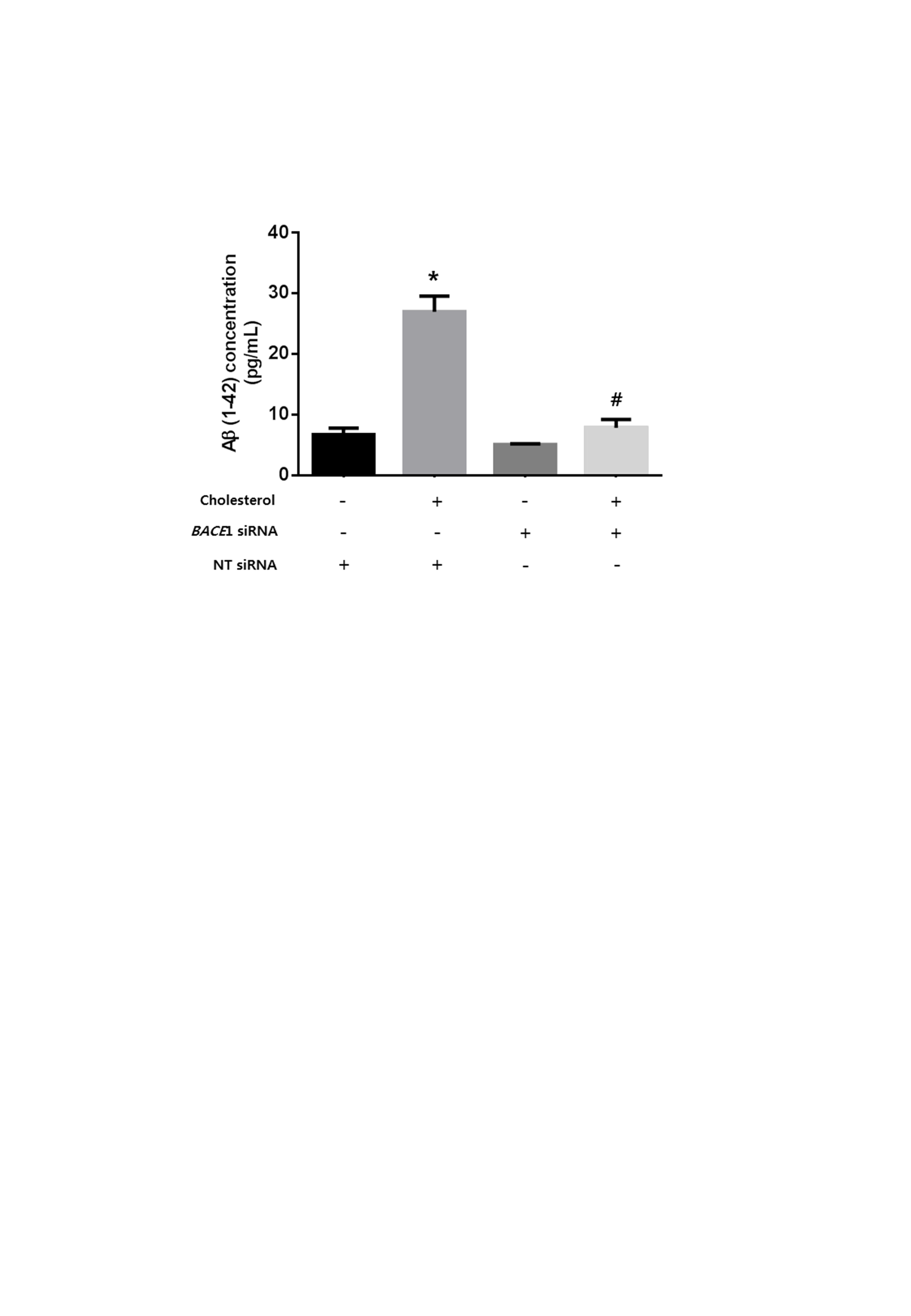

Supplement: Supplementary file 4 — Supplementary figure S2 [file 41419_2020_2663_MOESM4_ESM.tif]

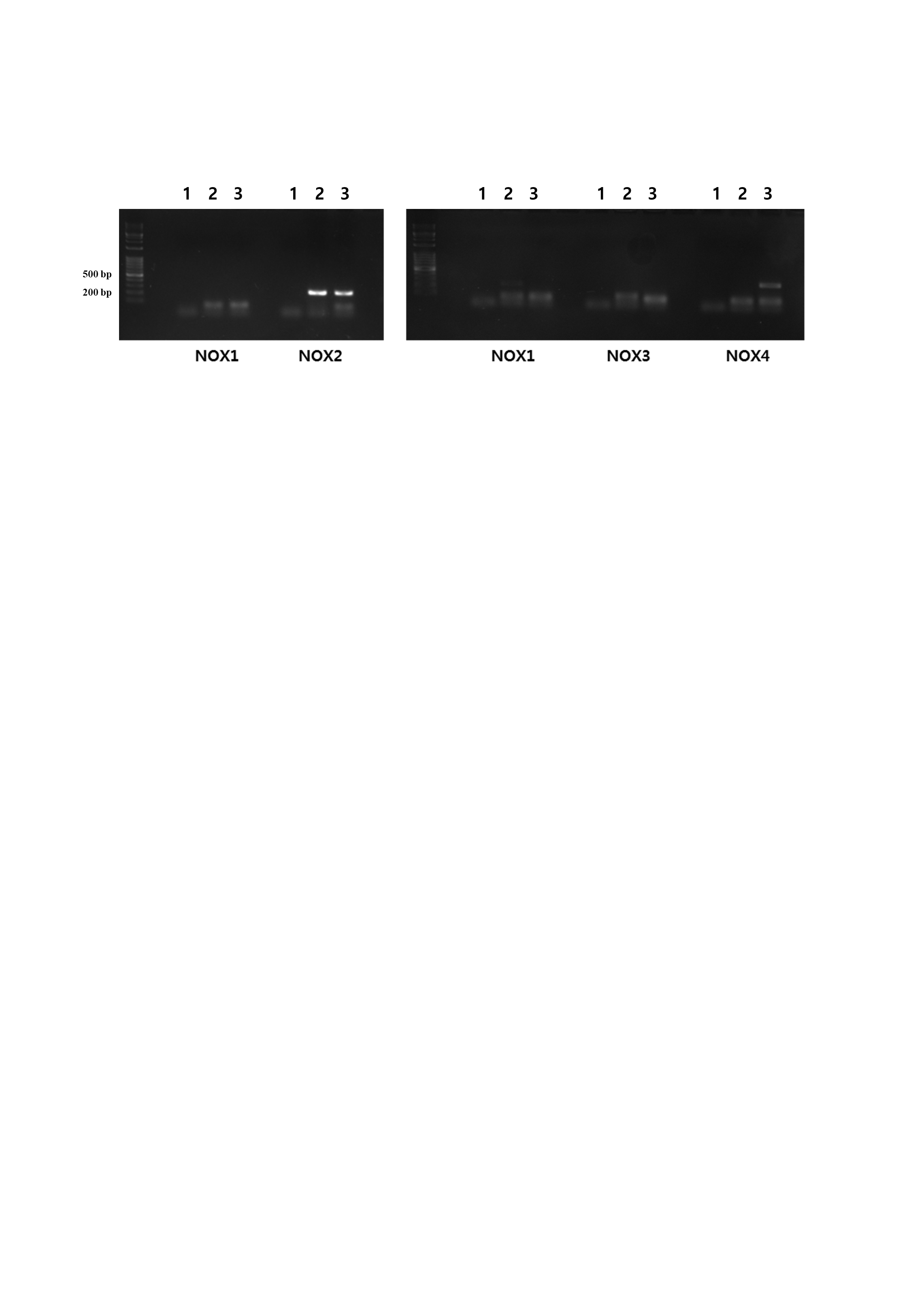

Supplement: Supplementary file 5 — Supplementary figure S3 [file 41419_2020_2663_MOESM5_ESM.tif]

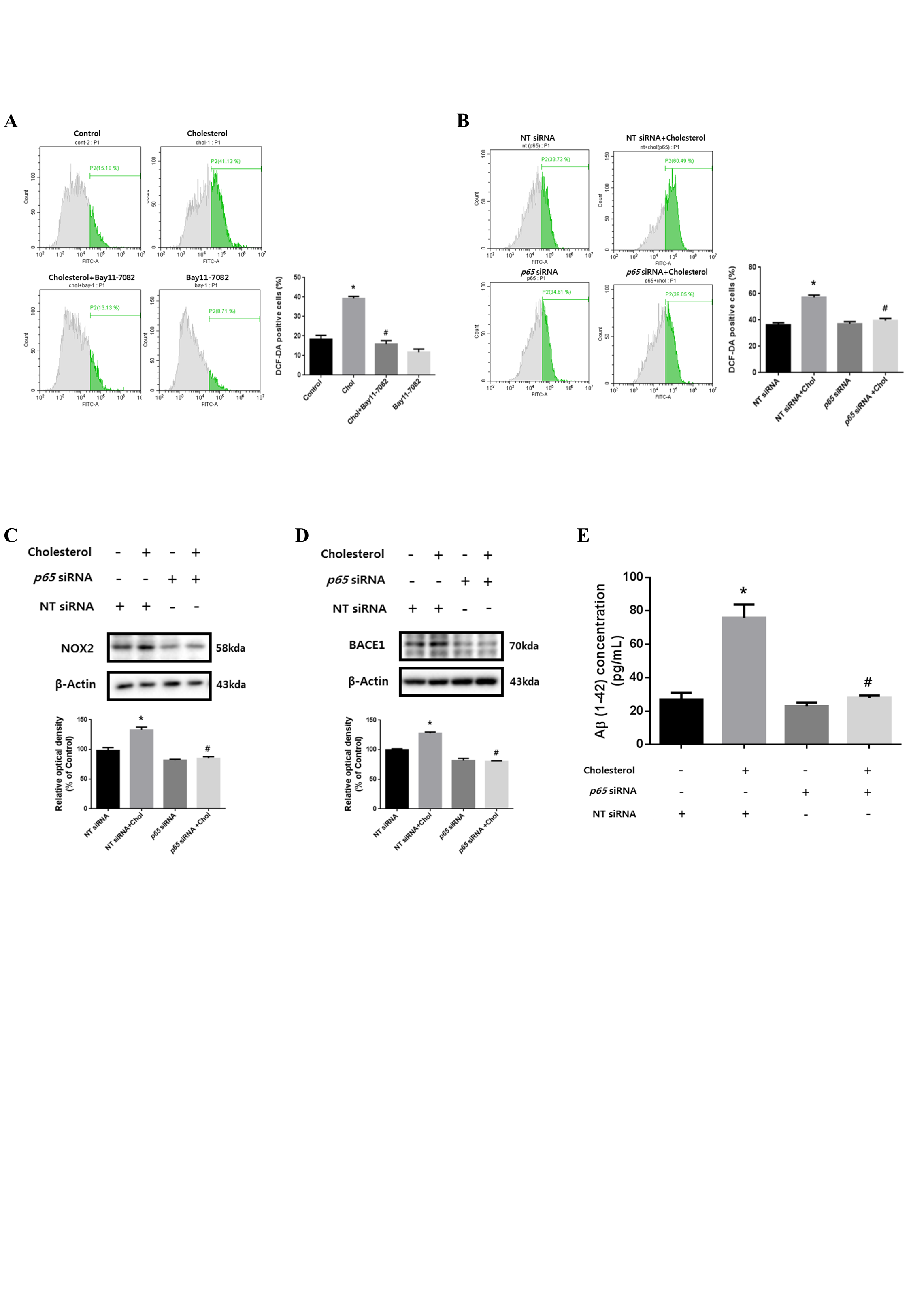

Supplement: Supplementary file 6 — Supplementary figure S4 [file 41419_2020_2663_MOESM6_ESM.tif]

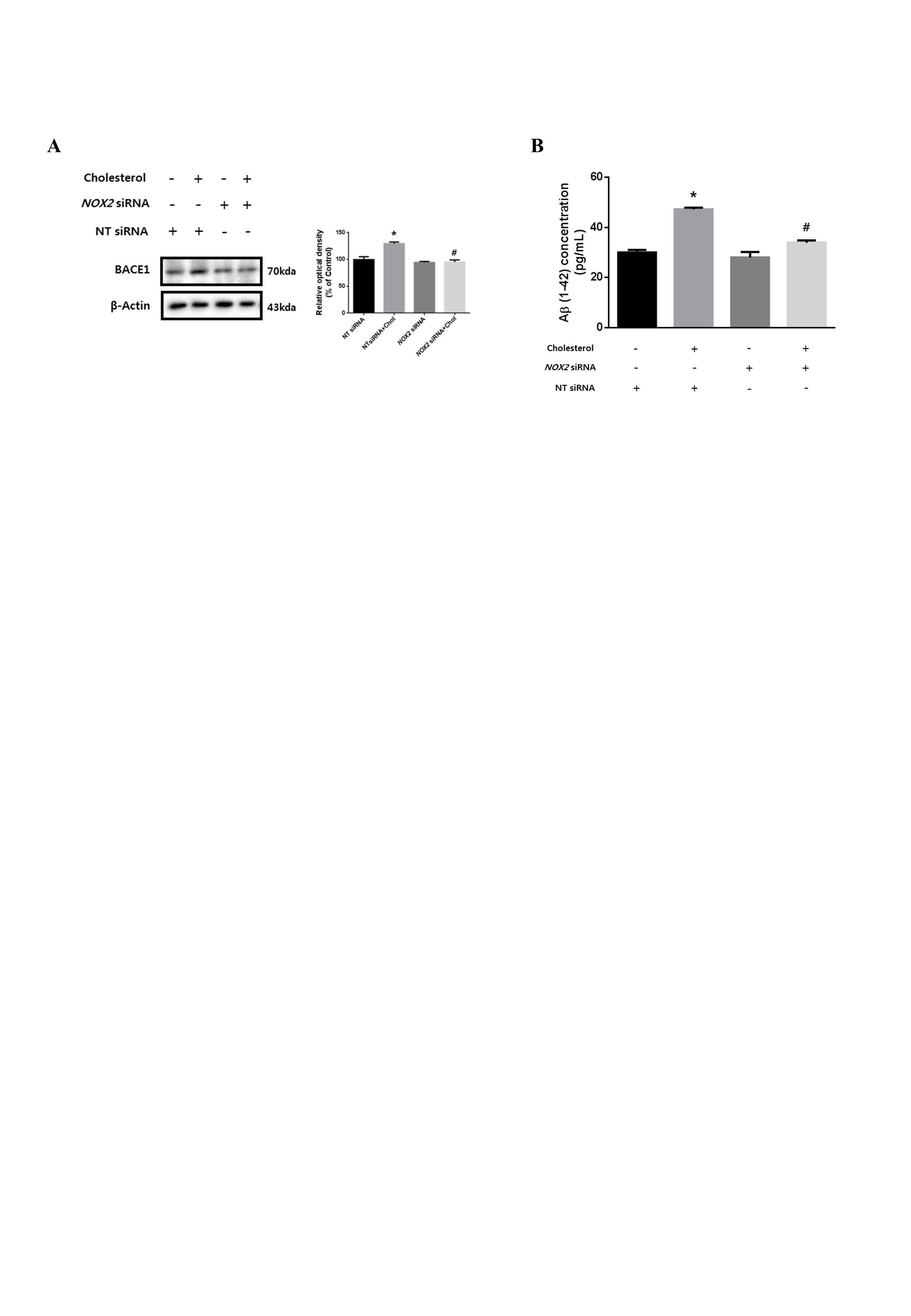

Supplement: Supplementary file 7 — Supplementary figure S5 [file 41419_2020_2663_MOESM7_ESM.tif]

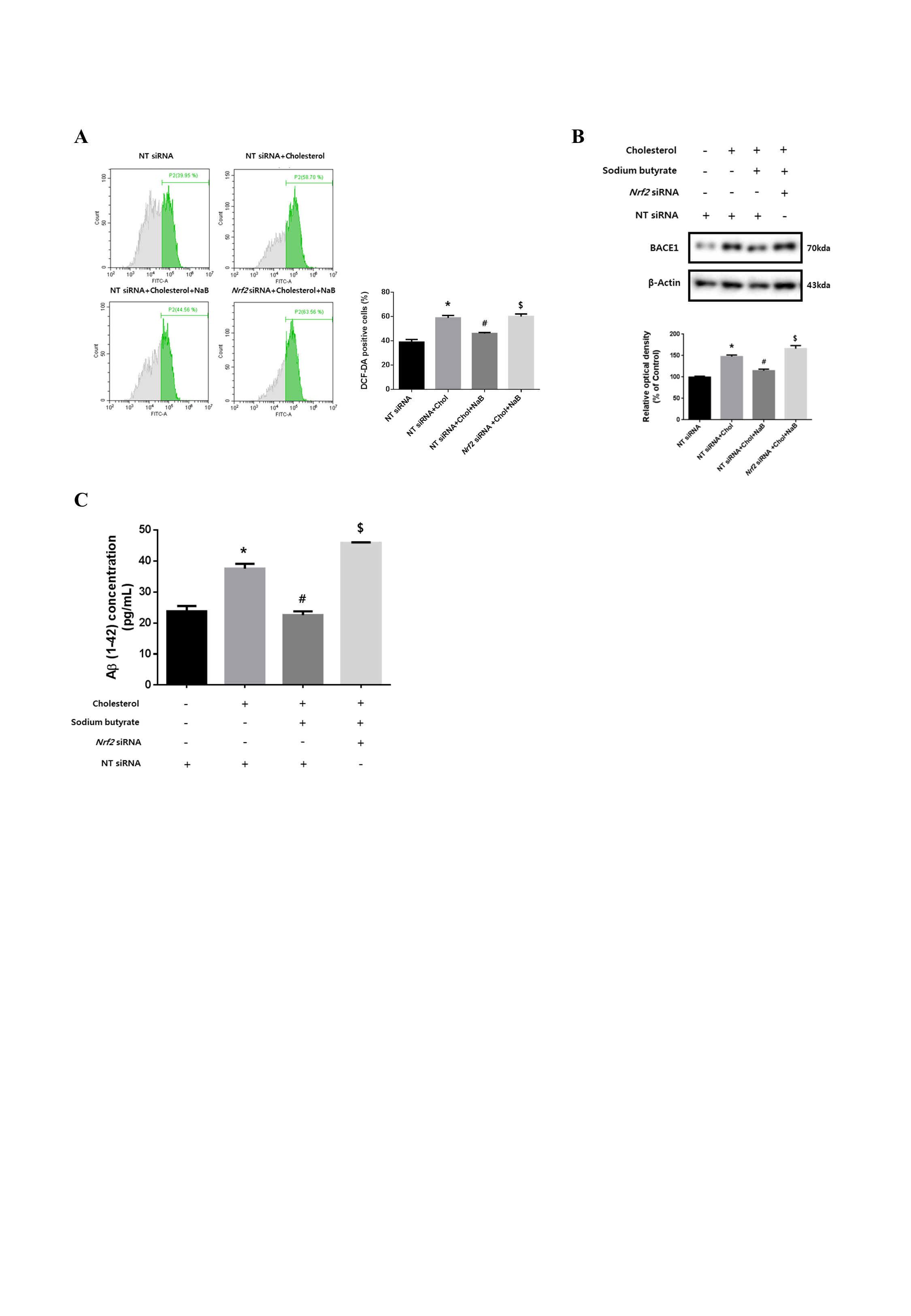

Supplement: Supplementary file 8 — Supplementary figure S6 [file 41419_2020_2663_MOESM8_ESM.tif]

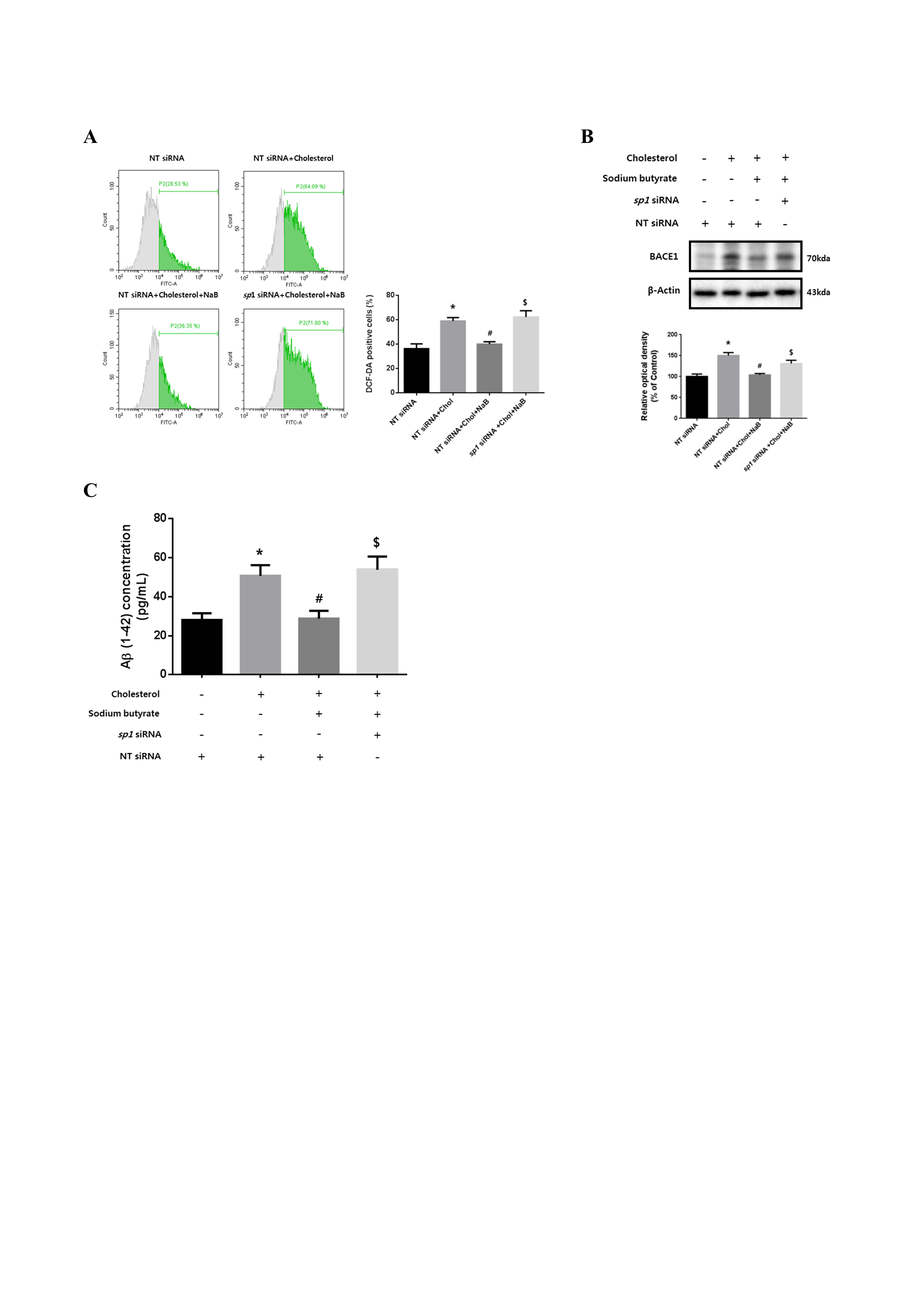

Supplement: Supplementary file 9 — Supplementary figure S7 [file 41419_2020_2663_MOESM9_ESM.tif]

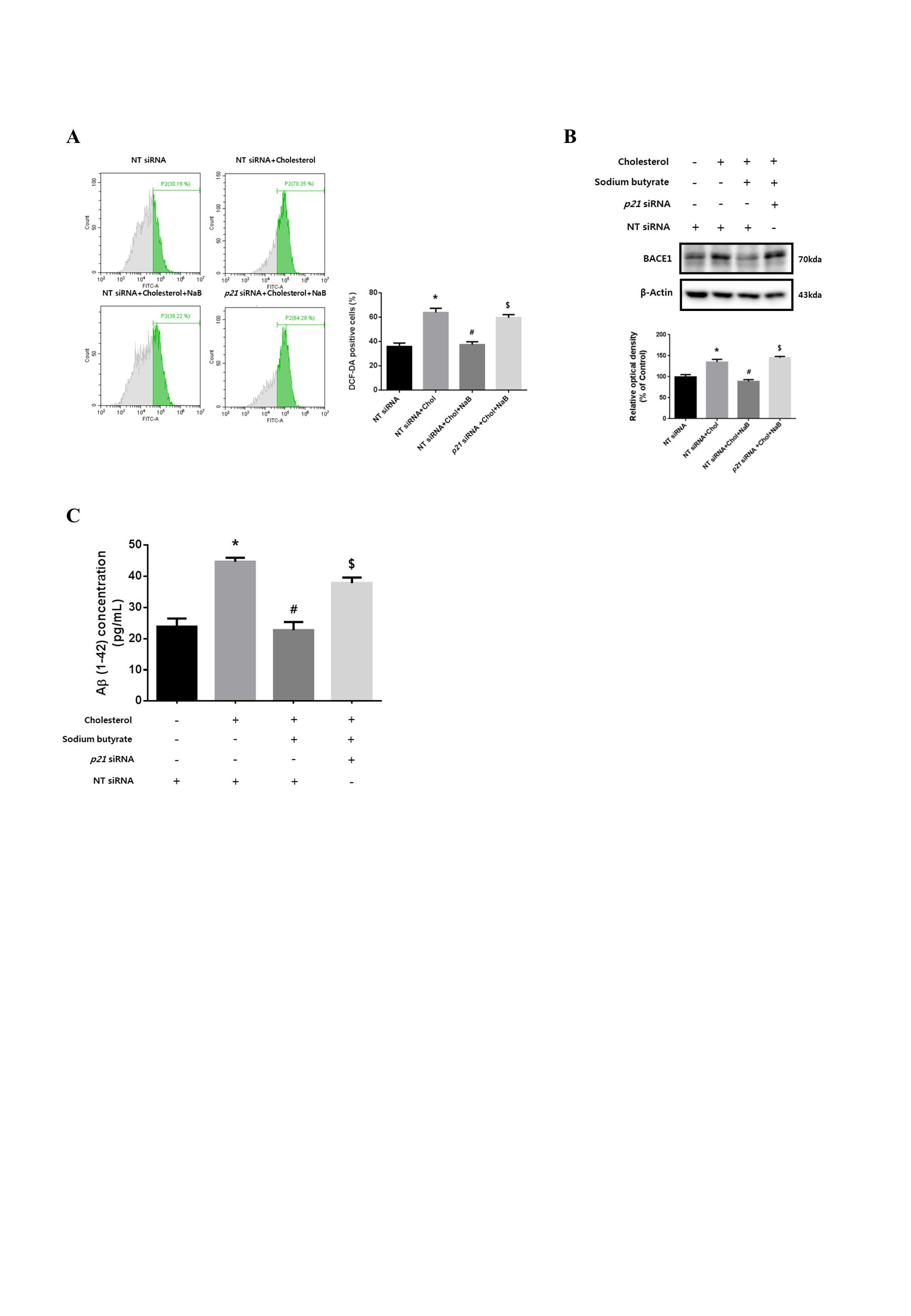

Supplement: Supplementary file 10 — Supplementary figure S8 [file 41419_2020_2663_MOESM10_ESM.tif]

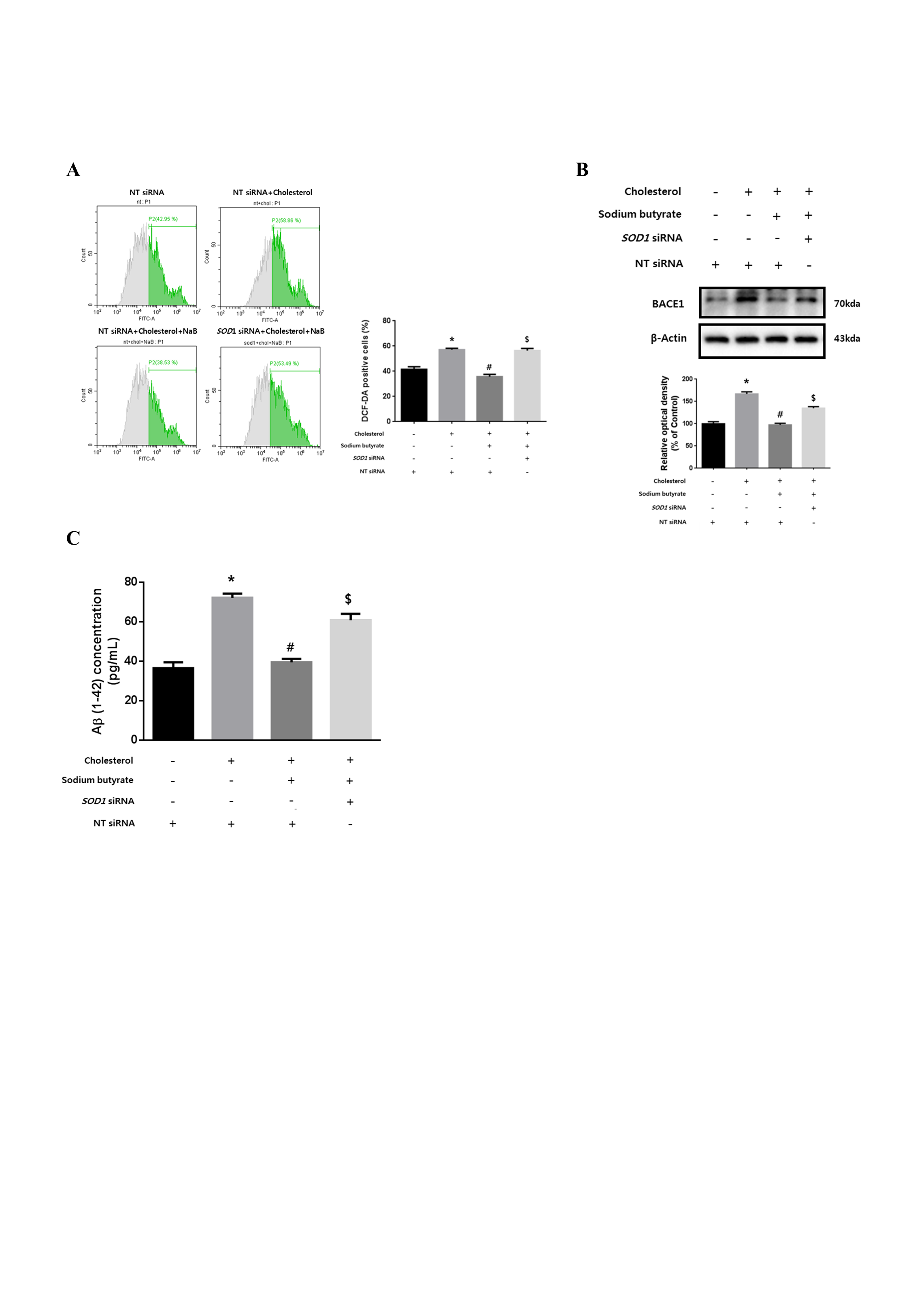

Supplement: Supplementary file 11 — Supplementary figure S9 [file 41419_2020_2663_MOESM11_ESM.tif]

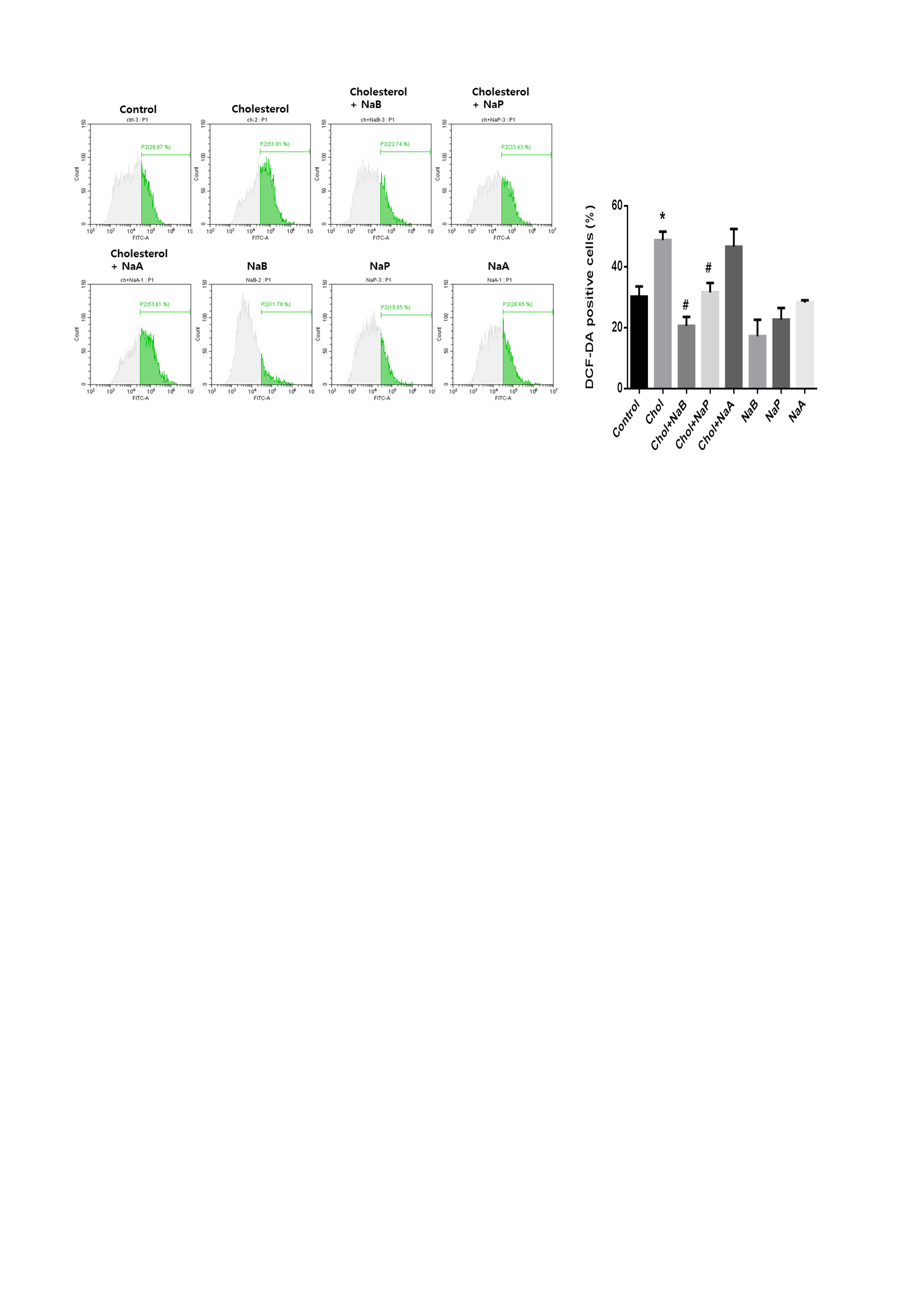

Supplement: Supplementary file 12 — Supplementary figure S10 [file 41419_2020_2663_MOESM12_ESM.tif]

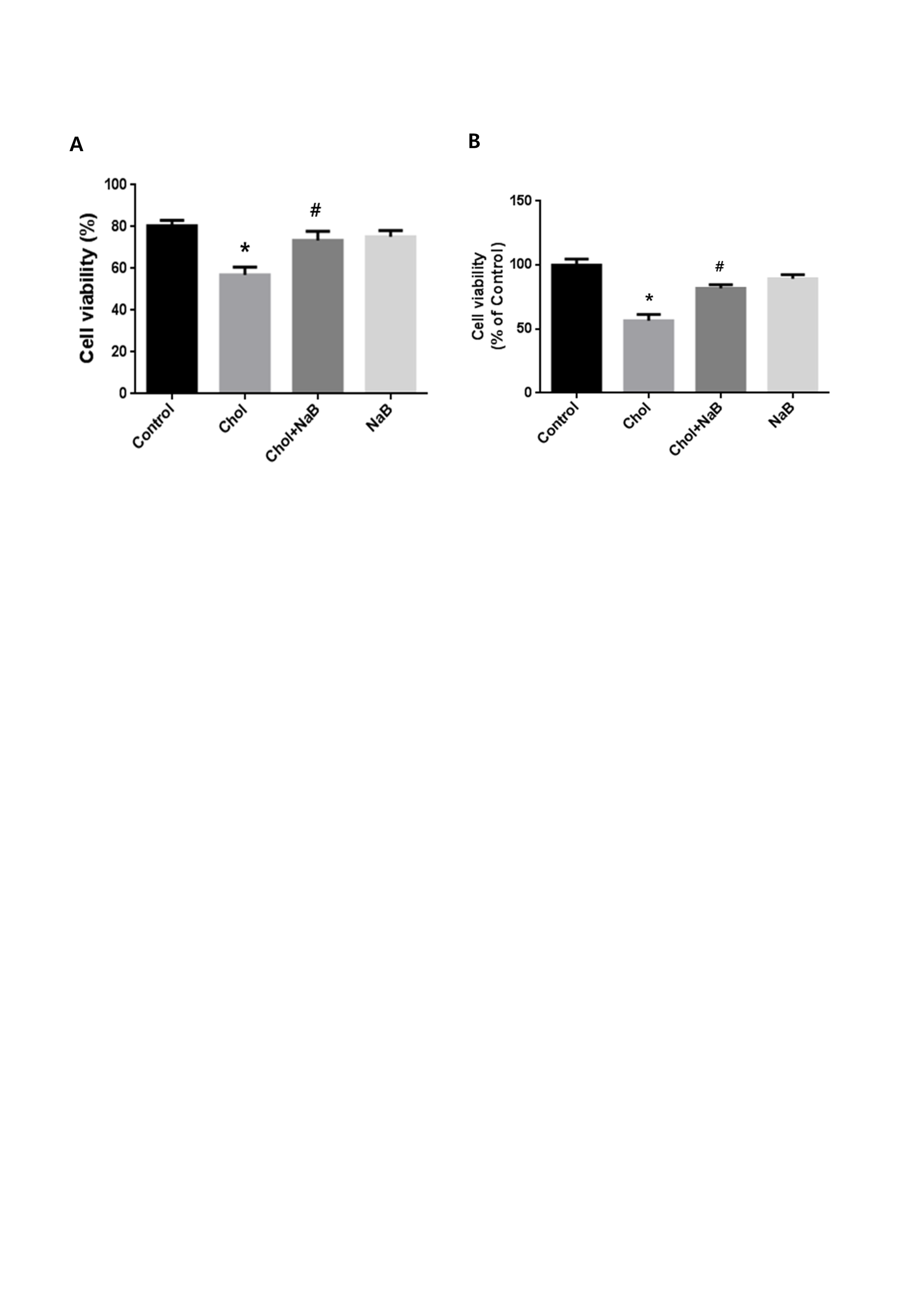

Supplement: Supplementary file 13 — Supplementary figure S11 [file 41419_2020_2663_MOESM13_ESM.tif]

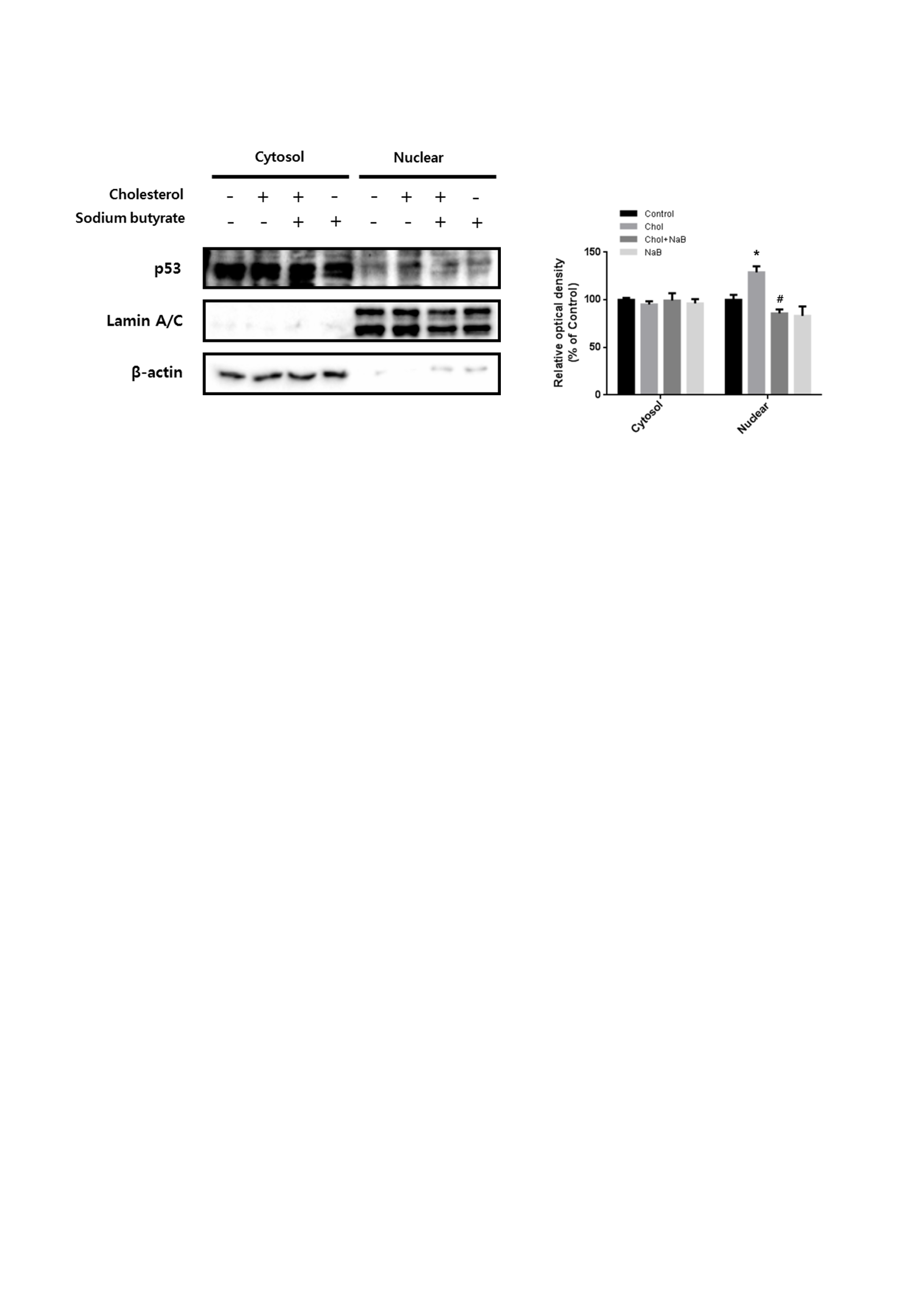

Supplement: Supplementary file 14 — Supplementary figure S12 [file 41419_2020_2663_MOESM14_ESM.tif]
